# Optimal Medical Therapy Targeting Lipids and Inflammation for Secondary Prevention in Patients Undergoing Percutaneous Coronary Intervention

**DOI:** 10.3390/jcm14238334

**Published:** 2025-11-24

**Authors:** Imma Forzano, Domenico Florimonte, Viviana Narciso, Mario Enrico Canonico, Domenico Simone Castiello, Lina Manzi, Stefano Cristiano, Alessandra Spinelli, Donato Maria Vallone, Dario D’Alconzo, Federica Semplice, Roberta Paolillo, Giuseppe Giugliano, Arturo Cesaro, Felice Gragnano, Paolo Calabrò, Giovanni Esposito, Giuseppe Gargiulo

**Affiliations:** 1 Department of Advanced Biomedical Sciences, Federico II University of Naples, 80131 Naples, Italy; imma.forzano@gmail.com (I.F.); florimontedomenico@gmail.com (D.F.); viviana.narciso@gmail.com (V.N.); marioenrico.canonico@unina.it (M.E.C.); ds.castiello@gmail.com (D.S.C.); lina.manzi93@gmail.com (L.M.); stefano.cristiano@unina.it (S.C.); alessandra.spinelli@unina.it (A.S.); donatovallone@outlook.it (D.M.V.); dario.dalconzo@gmail.com (D.D.); federicasemplic@gmail.com (F.S.); robe.paolillo@gmail.com (R.P.); giuseppe.giugliano@unina.it (G.G.); espogiov@unina.it (G.E.); 2 CPC Clinical Research, Department of Medicine, University of Colorado, Aurora, CO 80045, USA; 3 Department of Translational Medical Sciences, University of Campania “Luigi Vanvitelli”, 80138 Naples, Italy; arturo.cesaro@unicampania.it (A.C.); felice.gragnano@unicampania.it (F.G.); paolo.calabro@unicampania.it (P.C.); 4 Division of Cardiology, A.O.R.N. “Sant’Anna e San Sebastiano”, 81100 Caserta, Italy

**Keywords:** coronary artery disease, secondary prevention, percutaneous coronary intervention, acute coronary syndrome, chronic coronary syndrome, dyslipidemia, inflammation

## Abstract

Cardiovascular diseases (CVDs), including coronary artery disease (CAD), are the main causes of mortality and morbidity worldwide. The pathophysiology of CAD includes atherosclerosis, a chronic process leading to atherosclerotic plaque development. Clinical manifestations could be chronic, such as in the chronic coronary syndrome (CCS) scenario, or acute, such as acute coronary syndrome (ACS). The risk of subsequent cardiovascular (CV) events depends on the risk category defined by international guidelines. In particular, patients who have experienced a CV event requiring percutaneous coronary intervention (PCI) remain at heightened residual risk for subsequent events, despite advancements in standard-of-care strategies. Therefore, comprehensive residual risk management is essential in this population to mitigate ischemic risk. Secondary prevention includes different targets of treatments—hypertension, dyslipidemia, diabetes mellitus, body weight control, smoking habit reduction, and healthy lifestyle promotion. Nevertheless, there is a critical, unmet need for therapeutic strategies for this high-risk population. Growing evidence shows that atherogenic lipids and vascular inflammation drive residual risk after PCI, despite guideline-directed therapy. This review summarizes more recent evidence about secondary prevention focusing on optimal medical therapy (OMT), targeting lipids and inflammation for patients undergoing PCI.

## 1. Introduction

Cardiovascular diseases (CVDs), including coronary artery disease (CAD), are the principal cause of mortality and morbidity worldwide despite the improvement of invasive and non-invasive management [1]. CAD is mainly caused by atherosclerosis, a complex pathophysiological process characterized by lipid deposition and inflammation, leading to atherosclerotic plaque formation in the epicardial vessels [2]. Specifically, atherosclerosis is caused by the progressive deposition of low-density lipoprotein cholesterol (LDL-C) and other apolipoprotein-B (ApoB)-containing lipoproteins within the artery wall, which triggers a cascade of inflammatory responses determining the formation and progression of atherosclerotic plaque. It is well-established that LDL-C is not only a risk factor but also a direct cause of atherosclerosis; thus, lowering plasma LDL-C levels has become the main focus for preventing atherosclerotic CV events, for both primary and secondary prevention [3].

The CAD spectrum includes chronic coronary syndrome (CCS) and acute coronary syndrome (ACS). The European Society of Cardiology (ESC) guidelines define CCS as the clinical presentation of CAD during stable periods, including structural and/or functional alterations affecting the epicardial or microvascular compartment, causing hypoperfusion and ischemia. ACS refers to the acute event caused by the destabilization of atherosclerotic plaque with its complications, such as rupture, erosion, or thrombosis, leading to myocardial infarction (MI) [4].

**Figure 1:**
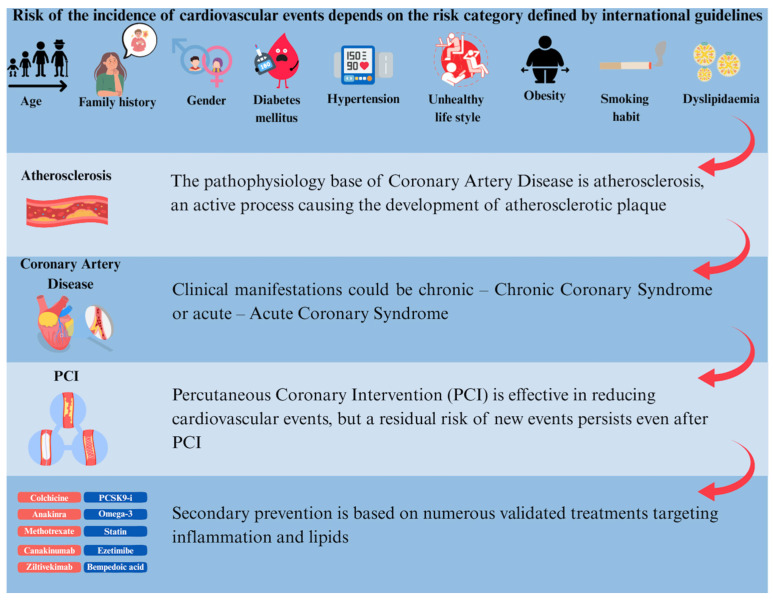
Atherosclerosis is a complex and chronic process leading to atherosclerotic plaque development. From atherosclerosis, CVDs develop, including CAD, which are the main causes of mortality and morbidity worldwide. Clinical manifestations of CAD could be chronic in the CCS scenario or acute such as ACS and could lead to PCI. Patients undergoing PCI need careful residual risk management to knock down the risk of a secondary event. There are effective and safe medical treatments that can be used in combination. ACS: Acute Coronary Syndrome; CAD: Coronary Artery Disease; CCS: Chronic Coronary Syndrome; CVD: Cardiovascular Disease; PCI: Percutaneous Coronary Intervention.

Medical therapy, including antithrombotic, anti-inflammatory, lipid-lowering, anti-diabetic, and anti-obesity drugs, is crucial to optimize clinical outcomes and prevent CV events. This review focuses on recent evidence on secondary prevention in patients with CAD, specifically focusing on lipids and inflammation as targets for secondary prevention in patients undergoing PCI, highlighting opportunities to improve prescription (Figure 1). Metabolic status will be addressed in a subsequent review, while antithrombotic therapy in secondary prevention is outside our scope.

### Residual Cardiovascular Risk

Medical therapy improves clinical outcomes in patients with CCS [5]. Coronary revascularization in patients affected by CAD demonstrated effects on symptoms, reducing the incidence of MI and CV death [6,7,8,9,10,11]. PCI is considered the standard of care in patients with ACS [12,13]. In CCS, the chosen modality of revascularization between coronary artery bypass graft (CABG) and PCI depends on multiple factors [14]. Secondary prevention after PCI is mandatory to reduce major adverse cardiovascular events (MACEs) [15]. Following PCI, patients remain at heightened residual risk for subsequent events, despite advancements in standard-of-care strategies [16]. The main determinants of CV residual risk include lipids, inflammation, an unhealthy lifestyle, metabolic diseases (such as diabetes and obesity), and thrombotic risk [17]. The residual CV risk is multifactorial, extending beyond revascularization. Antithrombotic therapy is fundamental in all patients undergoing PCI and reduces ischemic events [18,19,20,21,22,23,24,25,26,27,28]. However, it does not fully mitigate the long-term risk of MACEs. A subanalysis from the COMPASS (Cardiovascular Outcomes for People Using Anticoagulation Strategies) trial including patients with prior PCI demonstrated a 4% risk of MACEs at 3 years despite dual-pathway inhibition therapy with aspirin and low-dose rivaroxaban, and the risk was even higher (5%) in those receiving single antiplatelet therapy with aspirin alone [29]. High rates of MACEs were also observed in other studies evaluating antithrombotic therapies. In the TRITON–TIMI (Trial to Assess Improvement in Therapeutic Outcomes by Optimizing Platelet Inhibition with Prasugrel–Thrombolysis in Myocardial Infarction) 38 study, a MACE occurred in 12.1% of patients receiving clopidogrel and in 9.9% of those receiving prasugrel after 1.2 years of follow-up [30]. Similarly, in the PLATO (Platelet inhibition and patient Outcomes) trial, ischemic events, including death from vascular causes, MI, or stroke, occurred in 9.8% of patients treated with ticagrelor compared to 11.7% with clopidogrel at 1 year [31]. More recently, the ISAR-REACT (Rapid Early Action for Coronary Treatment) 5 study further confirmed the substantial risk of MACEs at 1 year, with event rates of 9.3% in the ticagrelor group and 6.9% in the prasugrel group [32]. Different trials have demonstrated that even with optimal antiplatelet regimens, residual risk remains largely driven by atherogenic dyslipidemia, particularly elevated levels of LDL-C, lipoprotein(a) (Lp-a), and triglyceride (TG)-rich lipo-proteins, as well as chronic metabolic disorders such as Type 2 Diabetes Mellitus (T2DM) and insulin resistance. A recent study conducted on 15,494 subjects investigated the impact of residual cholesterol and inflammatory risk in patients on statin therapy undergoing PCI. Intriguingly, it showed that residual inflammation, but not residual cholesterol risk, was associated with an increased risk of MACEs during 1-year follow-up, with a hazard ratio (HR) for inflammation at 1.78, 95% confidence interval (CI) of 1.36–2.33, and cholesterol HR at 1.01, 95% CI of 0.76–1.35 [33]. A study from the UK Biobank including 32,537 Atherosclerotic Cardiovascular Disease (ASCVD) patients demonstrated a higher risk of 1-year MACEs in those with elevated Lp(a) compared to normal values (41.2% vs. 35.6%; *p* < 0.001), with a consistent trend observed throughout the 5-year follow-up [34]. The residual CV risk was also considerable in studies assessing high-intensity lipid-lowering therapy (LLT) such as Proprotein Convertase Subtilisin/Kexin type 9 Inhibitors (PCSK9-i), and monoclonal antibodies, including evolocumab and alirocumab. In the FOURIER (Further Cardiovascular Outcomes Research With PCSK9 Inhibition in Subjects With Elevated Risk) trial, the risk of ischemic events, including CV death, MI, stroke, hospitalization for unstable angina, or coronary revascularization, remained high, with event rates of 9.8% in the evolocumab arm and 11.3% in the placebo arm at 48 weeks [35]. Similarly, in the ODYSSEY Outcomes (Evaluation of Cardiovascular Outcomes After an Acute Coronary Syndrome During Treatment With Alirocumab) trial, the incidence of the primary endpoint, including death from coronary heart disease, nonfatal MI, fatal or nonfatal ischemic stroke, or unstable angina requiring hospitalization, was 9.5% with alirocumab and 11.1% with placebo after 2.8 years of follow-up [36]. Hence, the residual CV risk is not solely attributable to lipid levels but also involves other concomitant factors, including persistent inflammation and endothelial dysfunction, leading to atherosclerotic progression and its complications [37]. The CANTOS (Canakinumab Anti-Inflammatory Thrombosis Outcomes Study) trial investigated the relationship between inflammation and atherosclerosis comparing canakinumab, an interleukin-1β (IL-1β) inhibitor, versus placebo. The study enrolled patients with prior MI and elevated high-sensitivity C-reactive protein (hsCRP) (≥2 mg/L) [38]. More than 90% of patients at baseline were treated with guideline-directed medical therapy (GDMT) including LLT, antithrombotic, and anti-ischemic therapies. The incidence of MACEs was 4.50 events per 100 patient-year in the placebo group, compared with 4.11, 3.86, and 3.90 events per 100 person-year in the 50 mg, 150 mg, and 300 mg canakinumab groups, respectively. HRs comparing canakinumab versus placebo were 0.93 (95% CI, 0.80–1.07) for the 50 mg dose, 0.85 (95% CI, 0.74–0.98) for the 150 mg dose, and 0.86 (95% CI, 0.75–0.99) for the 300 mg dose [38]. The study demonstrated that canakinumab 150 mg significantly reduced MACEs independent of lipid levels, confirming inflammation as a modifiable causal factor of atherothrombosis and highlighting the importance of residual inflammatory risk, which encompasses the risk of CV events in patients who achieved optimal LDL-C levels and concomitant elevated markers of inflammation, such as hsCRP [39]. Atherosclerosis is not solely driven by lipids but also by inflammation. Data from the PROVE-IT (Pravastatin or Atorvastatin Evaluation International Trial) and IMPROVE-IT (Improved Reduction of Outcomes: Vytorin Efficacy International Trial) studies demonstrated that achieving both low LDL-C and low hsCRP levels results in greater CV risk reduction [39]. Nevertheless, real-world data show the suboptimal prescription of guideline-directed medical therapy (GDMT) in patients affected by CAD [40,41,42]. Altogether, this highlights the need for a comprehensive, multi-targeted approach combining LLT, metabolic control, anti-inflammatory, and antithrombotic strategies to reduce residual CV risk after PCI.

## 2. Pathophysiology

### 2.1. Pathophysiology of Lipids

Lipids are essential to cellular integrity, energy storage, and signaling. TG serves as a principal energy source, cholesterol maintains membrane structure and supports steroid synthesis, and lipoproteins ensure lipid transport within the circulation. Lipid metabolism occurs through two major pathways: exogenous and endogenous. In the exogenous route, dietary lipids absorbed by enterocytes are incorporated into chylomicrons, which enter the lymphatic system and subsequently the bloodstream. Chylomicrons deliver TG to peripheral tissues via lipoprotein lipase (LPL), becoming cholesterol-rich remnants that are cleared by the liver. The endogenous pathway begins in the liver, where very low-density lipoproteins (VLDLs) are synthesized to transport endogenously produced TG and cholesterol. Sequential lipolysis of VLDLs generates intermediate-density lipoproteins (IDLs) and, eventually, LDL-C, the main carriers of cholesterol to peripheral tissues. High-density lipoproteins (HDLs) mediate reverse cholesterol transport, removing excess cholesterol from tissues back to the liver [43].

Excess circulating LDL-C is central to the pathogenesis of atherosclerosis. ApoB-containing lipoproteins smaller than 70 nm, including LDL, VLDL remnants, IDL, and Lp(a), penetrate the arterial intima in proportion to plasma concentration. Retained lipoproteins bind to intimal proteoglycans and undergo oxidative modification by NADH/NADPH oxidases. Oxidized LDL-C is internalized by macrophage scavenger receptors, forming foam cells and fatty streaks, the earliest lesions of atherosclerosis. This process triggers the recruitment of inflammatory cells and release of cytokines that stimulate smooth muscle cell (SMC) proliferation and extracellular matrix deposition, contributing to mature atherosclerotic plaque formation. Ultimately, inflammatory cells can lead to plaque destabilization through apoptosis of SMC, endothelial cells, and macrophages [44].

LDL-C is the principal therapeutic target in LLT. According to current ESC guidelines, patients with established ASCVD should achieve LDL-C <1.4 mmol/L (<55 mg/dL), or <1.0 mmol/L (<40 mg/dL) if they experience recurrent vascular events or in case of polyvascular arterial disease [3,12,16]. However, large registries such as the EUROASPIRE (European Action on Secondary and Primary Prevention by Intervention to Reduce Events) V, ICLPS (International ChoLesterol management Practice Study), and DA VINCI (Do stAtins faVourably modify atherosclerotIc plaque in patients with differeNt levels of polygenic Cardiovascular rIsk?) studies reveal substantial gaps in target achievement, with less than one-third of very-high-risk patients reaching recommended LDL-C levels [45,46,47].

HDL, conversely, exerts atheroprotective effects through reverse cholesterol transport, anti-inflammatory, and antioxidant mechanisms [48]. Yet, despite strong epidemiologic associations, Mendelian randomization and interventional trials have failed to demonstrate a causal or therapeutic benefit from HDL-raising strategies, underscoring the complexity of HDL functionality [49,50].

Hypertriglyceridemia is increasingly recognized as an independent contributor to atherogenesis [3,16]. While large TG-rich lipoproteins such as chylomicrons and VLDL cannot cross the endothelium, their remnants, the smaller TG-rich lipoproteins, are thought to be equally or more atherogenic than LDL-C. They can penetrate and become retained within the subendothelial space through electrostatic interactions between apolipoprotein B and proteoglycans. TGRL remnants deliver more cholesterol per particle than LDL-C and induce oxidative stress, cytokine release (TNF-α, IL-1β), endothelial dysfunction, and coagulation activation via upregulation of plasminogen activator inhibitor-1, collectively fostering inflammation and thrombosis [51,52]. Mendelian randomization studies support a causal role of remnant cholesterol in both inflammation and ischemic heart disease, highlighting that all ApoB-containing lipoproteins contribute similarly to ASCVD risk [53].

Lp(a) is an LDL-C particle with an Apo(a) protein covalently bound to its ApoB component [54]. It is <70 nm in diameter and can freely flux across the endothelial barrier, with a similar action to LDL-C, and may have pro-atherogenic effects. Its role in increasing CV risk has also been attributed to pro-coagulant effects, given the similar structure of plasminogen. The pro-inflammatory effects of Lp(a) are most likely related to the oxidized phospholipid [55]. Elevated Lp(a) levels are largely genetically determined and confer a lifetime ASCVD risk comparable to that observed in heterozygous familial hypercholesterolemia when exceeding 180 mg/dL (430 nmol/L) [56]. Although modest Lp(a) reductions with niacin or CETP inhibitors failed to improve outcomes, emerging data suggest that the more profound reductions achievable with PCSK9 inhibitors may confer incremental benefit [57]. Mendelian randomization analyses confirm a causal relationship between Lp(a) concentration and ASCVD risk, proportional to the absolute degree of elevation 92. For these reasons, the recent update to the ESC guidelines about the management of dyslipidemias considers Lp(a) as a CV-risk enhancing factor, with a linear association between its levels and CV risk, from a concentration of 50 mg/dl (150 nmol/L). Furthermore, Lp(a) assessment should be considered at least once in every adult’s lifetime [58].

Finally, through IntraVascular Ultrasound (IVUS) and Near-Infrared Spectroscopy (NIRS) assessment, the PROSPECT (Identification of vulnerable plaques and patients by intracoronary near-infrared spectroscopy and ultrasound) II study demonstrated that elevated LDL-C, TG, and Lp(a) levels are closely associated with lipid-rich plaques, while high Lp(a) particularly predicts focal plaque vulnerability [59,60].

These findings reinforce the multifaceted role of lipoproteins, not only in the initiation and progression of coronary atherosclerosis but also in determining plaque instability and clinical outcomes, leading to the conclusion that LLT represents one of the most impactful therapeutic arms for the treatment of atherosclerotic diseases.

### 2.2. Pathophysiology of Inflammation

The inflammatory nature of atherosclerosis, first proposed by Rudolph Virchow in the 19th century, was empirically validated only a century later. In the 1980s, Goran and colleagues demonstrated, through immunofluorescence studies of carotid plaques, a dense accumulation of macrophages and monocytes within necrotic cores, together with CD3^+^ T cells and, to a lesser extent, B cells in the fibrous cap and shoulder regions. Importantly, they identified the strong expression of HLA-DR, an MHC class II antigen, in both immune cells and SMCs, revealing not only immune cell activity but also the potential for SMC phenotypic modulation, and establishing inflammation as a central mechanism in atherogenesis. Subsequent experimental work confirmed this concept: hyperlipidemic mice lacking T and B cells developed markedly less atherosclerosis than immunocompetent counterparts, highlighting the critical role of adaptive immunity in plaque formation [61].

Inflammation drives every phase of atherosclerosis, from the earliest endothelial dysfunction to plaque rupture and thrombosis. Clinical evidence has reinforced this paradigm. A pooled analysis of three randomized trials (PROMINENT, REDUCE-IT, and STRENGHT) showed that hsCRP, a biomarker of systemic inflammation, predicted cardiovascular events more strongly than LDL-C levels in statin-treated patients [62]. Likewise, persistent inflammation despite LLT has been linked to residual cardiovascular risk in patients undergoing PCI [33].

Some studies have reported IL-1ꞵ and IL-6 as promoting factors in atherosclerosis. Moreover, inflammation status is responsible for increasing soluble inflammatory mediators that perpetuate the activation of inflammatory pathways, playing a significant role in the progression of the disease [63].

Among the key mediators, IL-1β and interleukin-6 (IL-6) are pivotal in sustaining vascular inflammation [63]. Modified lipids internalized by macrophages activate the NLRP3 inflammasome, leading to caspase-1 activation and cleavage of pro-IL-1β into its mature form. IL-1β promotes endothelial adhesion of leukocytes, SMC proliferation, and vessel remodeling, while simultaneously stimulating IL-6 release, amplifying the inflammatory cascade [64]. Experimental inhibition or genetic deletion of NLRP3 and IL-6 reduces atherosclerotic burden, supporting its causal role in plaque development [65].

IL-6 is markedly upregulated, by up to 40-fold, in atherosclerotic lesions compared to healthy vessels. Through JAK/STAT signaling, IL-6 enhances hepatic synthesis of CRP and fibrinogen, activates endothelial cells, and promotes leukocyte recruitment. It also increases macrophage expression of LDL receptors and monocyte chemoattractant protein-1 (MCP-1), augmenting foam cell formation. Furthermore, IL-6 upregulates vascular cell adhesion molecule-1 (VCAM-1) and intercellular adhesion molecule-1 (ICAM-1), facilitates T-cell differentiation, and enhances angiotensin II type 1 receptor expression in SMCs, exacerbating oxidative stress and endothelial dysfunction. By inducing matrix metalloproteinases (MMPs) and plasminogen activator inhibitor-1 (PAI-1), IL-6 contributes to extracellular matrix degradation, plaque instability, and thrombosis. A self-amplifying feedback loop driven by IL-6–induced CD44 overexpression perpetuates this chronic inflammatory state, ultimately linking vascular inflammation to the clinical manifestations of coronary atherosclerosis [66].

Considering these findings, with a pivotal role of acute and persistent inflammation in the pathogenesis of atherosclerosis, there is growing evidence surrounding anti-inflammatory therapies for secondary cardiovascular prevention [67].

## 3. Secondary Prevention

### 3.1. Targeting Lipids

#### 3.1.1. Statin, Ezetimibe, and Combination Therapy

**Figure 2:**
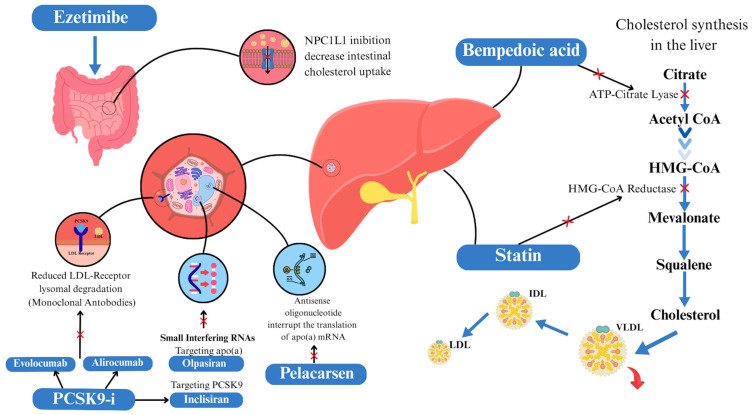
Focus on the mechanism of action of cholesterol-lowering drugs. Statins inhibit the synthesis of endogenous cholesterol by acting on the enzyme HMG Co-A reductase, which converts the 3-hydroxy-3-methylglutaryl-CoA molecule into mevalonate, a precursor of cholesterol. The reduction in intracellular cholesterol increases the expression of LDL-R on the surface of hepatocytes, enhancing the uptake of LDL-C from the bloodstream and lowering plasma concentrations of LDL-C and other ApoB-containing lipoproteins. Ezetimibe reduces cholesterol absorption through interaction with the NPC1L1 included in the small intestine. This causes a reduction in LDL-C transit in the liver and the increased expression of LDL-R on liver cell surfaces with a reduction in LDL-C levels due to the increased uptake of LDL-C in the cells. PCSK9-i can be monoclonal antibodies (alirocumab, evolocumab) or a small interfering ribonucleic acid (inclisiran) that target PCSK9 in the liver. PCSK9 promotes the degradation of LDL-C receptors on the hepatic cell surface. By inhibiting PCSK9, these agents increase the availability of LDL-R, thereby enhancing LDL-C clearance and significantly reducing circulating LDL-C levels. Bempedoic acid inhibits ATP citrate lyase, reducing acetyl CoA formation and reducing cholesterol levels. Olpasiran is a small interfering RNA presently under investigation that was demonstrated to reduce Lp(a) synthesis in the liver. Pelacarsen is a hepatocyte-directed antisense oligonucleotide demonstrated to reduce Lp(a) through interruption of the translation of apo(a) mRNA. Apo: Apolipoprotein; HMG Co-A: Hydroxymethylglutaryl-CoA; IDL: Intermediate-Density Lipoprotein; LDL-C: Low-Density Lipoprotein; LDL-R: Low-Density Lipoprotein Receptor; NPC1L1: Niemann–Pick C1-like protein 1; PCSK9-i: Proprotein Convertase Subtilisin/Kexin type 9 Inhibitor; VLDL: Very-Low-Density Lipoprotein, Lp(a): Lipoprotein(a). x indicates inhibition by the drug.

Statins are the cornerstone of LLT in patients undergoing PCI (Figure 2). Statins inhibit the synthesis of endogenous cholesterol by acting on the enzyme hydroxymethylglutaryl-CoA (HMG Co-A) reductase, which converts the 3-hydroxy-3-methylglutaryl-CoA molecule into mevalonic acid, a precursor of cholesterol. The reduction in intracellular cholesterol increases the expression of LDL-R on the surface of hepatocytes, enhancing the uptake of LDL-C from the bloodstream and lowering plasma concentrations of LDL-C and other ApoB-containing lipoproteins, including TGRLs [68].

The lipid-lowering effect occurs in a dose-dependent manner, with the magnitude of reduction determined by the intrinsic potency of the statin. A high-intensity regimen is defined as the dose of a statin that, on average, reduces LDL-C plasma levels by ≥50%, regardless of interindividual variation in LDL-C reduction [69,70,71].

Statins also reduce TG levels by 10–20% [72]. The mechanism of the TG-lowering effect has not been fully elucidated, but it seems to be partly independent from the classic HMG-CoA pathway. It may involve the upregulation of VLDL uptake by hepatocytes, as well as a reduction in the production rate of VLDL. Statins seem to have other beneficial effects, so-called pleiotropic effects [73]. Among these, the most notable are the anti-inflammatory and antioxidant actions that result in atherosclerotic plaque stabilization and endothelial function improvement. These effects have been shown in vitro and in experimental systems, but their clinical relevance remains unproven [74,75]. In the specific setting of PCI patients, statins have also been demonstrated to reduce the risk of contrast-induced acute kidney injury [76]. The benefits of statins in reducing CV risk is mainly driven by lowering plasma LDL-C levels [77,78,79]. As discussed before, patients undergoing PCI are at a very high risk of ASCVD, and statin therapy concurs to mitigate the ischemic risk.

Several studies have shown that early use of statins in patients with ACS provides significant benefits in reducing the recurrence of MACEs [80,81,82,83,84,85]. The SECURE PCI (Statins Evaluation in Coronary Procedures and Revascularization) randomized, placebo-controlled trial assessed the impact of peri-procedural loading with atorvastatin (i.e., two loading doses of 80 mg, before and 24 h after the planned PCI) on MACEs at 30 days in 4191 patients with ACS and planned invasive management, compared to placebo [85]. All patients received atorvastatin 40 mg/die starting 24 h after the second loading dose. The authors found no significant treatment benefit in the overall study population [at 30 days: in the atorvastatin group, 130 patients had a MACE (6.2%), and in the placebo group 149 patients had a MACE (7.1%), with an absolute difference of 0.85%; 95% CI, −0.70% to 2.41%; HR, 0.88; 95% CI, 0.69–1.11; *p* = 0.27)]. In a pre-specified analysis, a significant 28% relative risk reduction in MACEs was observed among patients who underwent PCI (65% of all patients). The benefit was even more pronounced (46% relative risk reduction) in a post hoc analysis including 865 ST-elevation MI (STEMI) patients undergoing primary PCI. In a meta-analysis of randomized trials including individual data from 3341 patients undergoing PCI, both pre-treatment with high-intensity statins in statin-naïve patients and statin loading in those already on therapy significantly reduced the risk of peri-procedural MI and 30-day CV events by over 40%. Event rates were 7.0% vs. 11.9% (*p* < 0.0001) for statin-naïve patients and 7.4% vs. 12.6% (*p* = 0.05) for those already on statins [86]. PCI was performed in the setting of stable angina or in a non-emergency setting in Non-ST-elevation MI (NSTEMI). One of the studies that was included in the meta-analysis showed an improvement in coronary flow when primary PCI was used for the treatment of STEMI [87].

The LIPS (Lescol Intervention Prevention Study) study demonstrated that treatment with fluvastatin 80 mg/day led to significant reductions in MACEs following PCI in patients with stable and unstable angina with average cholesterol levels. Fluvastatin reduced the risk of MACEs by 28% compared with placebo (*p* = 0.03) among patients with unstable angina; no difference between patients with stable and patients with unstable angina was observed (relative risk 1.07, 95% CI 0.87 to 1.30, *p* = 0.53); and fluvastatin reduced MACEs, excluding restenosis, by 36% (*p* = 0.006) among patients with unstable angina and 31% (*p* = 0.02) among patients with stable angina [88].

Patients undergoing PCI, according to ESC guidelines, need to achieve LDL-C plasma levels <1.4 mmol/L (<55 mg/dL) and a reduction by at least 50% from baseline. For patients who experience a second CV event within 2 years while taking maximum tolerated statin-based therapy, an even lower LDL-C goal of <1.0 mmol/L (40 mg/dL) may be considered [4]. It is therefore a rational approach to start directly with high-intensity statin therapy to reach this target. The SPACE ROCKET (Secondary Prevention of Acute Coronary Events—Reduction Of Cholesterol to Key European Targets) Trial Group has shown that rosuvastatin (high-intensity statin) reduced LDL-C levels more effectively than simvastatin (moderate-intensity statin) in patients with hyperlipidemia and acute MI, reaching the recommended target in a greater percentage of patients (OR: 1.16; 95% CI: 0.88–1.53; *p* = 0.29) [89]. Similarly, the PROVE-IT (Pravastatin or Atorvastatin Evaluation International Trial) trial enrolled 4162 patients who had been hospitalized for an ACS within 10 days comparing 40 mg of pravastatin daily (standard therapy) versus 80 mg of atorvastatin daily (intensive therapy). The primary end point was a composite of death from any cause, MI, documented unstable angina requiring rehospitalization, revascularization (performed at least 30 days after randomization), and stroke. Follow-up lasted 18 to 36 months (mean, 24). Kaplan–Meier estimates of the rates of the primary end point at two years were 26.3% in the pravastatin group and 22.4% in the atorvastatin group, reflecting a 16% relative reduction in the risk in favor of atorvastatin (95% CI, 5–26%, *p* = 0.005). The study did not meet the prespecified criterion for equivalence but did identify the superiority of the more intensive regimen [90].

The IDEAL (High-Dose Atorvastatin vs. Usual-Dose Simvastatin for Secondary Prevention after Myocardial Infarction) study showed that, in patients with a history of MI, intensive LDL-C lowering did not significantly reduce the primary outcome of the MACEs (HR 0.89; 95% CI, 0.78–1.01; *p* = 0.07). However, it was associated with a reduction in certain secondary composite endpoints, including nonfatal acute MI. No differences were observed in CV or all-cause mortality [91].

Ezetimibe reduces cholesterol absorption through interaction with the Niemann–Pick C1-like protein 1 (NPC1L1) included in the small intestine. This causes reduction in LDL-C transit in the liver and an increased expression of LDL-R on liver cell surfaces, with a reduction in LDL-C levels due to the increased uptake of LDL-C in the cells [92]. Ezetimibe was demonstrated to be safe and effective in reducing LDL-C blood levels [93,94,95]. In monotherapy, ezetimibe was demonstrated to reduce LDL-C levels by about 19% [94].

IMPROVE-IT (Improved Reduction of Outcomes: Vytorin Efficacy International Trial) was a randomized clinical trial involving 18,144 patients hospitalized for ACS, which demonstrated that combination therapy with simvastatin (40 mg) and ezetimibe, compared to simvastatin plus placebo, led to further LDL-C reduction and improved CV outcomes (absolute risk difference, 2.0 percentage points; HR 0.936; 95% CI, 0.89 to 0.99; *p* = 0.016) without an increase in adverse events [96].

In the RACING (Randomized Controlled Trial of Moderate-intensity Statin with Ezetimibe Combination Therapy) trial, it was discovered that in patients with ASCVD, moderate-intensity statin with ezetimibe combination therapy was not inferior to high-intensity statin monotherapy. The primary endpoint occurred in 172 patients (9.1%) in the combination therapy group and in 186 patients (9.9%) in the high-intensity statin monotherapy group with an absolute difference of −0.78% (90% CI −2.39 to 0.83). There was a lower intolerance-related drug discontinuation with a higher rate of patients who reached LDL-C <70 mg/dL. The large CONNECT DES (A Whole Population-based Study on COreaN NationwidE Claims daTa on Drug-Eluting Stent) registry evaluated that in clinical practice, LLT with ezetimibe and moderate-intensity statins was associated with favorable clinical outcomes and drug compliance in patients who underwent PCI with drug eluting stent (DES) implantation. In particular, with combination LLT, a lower occurrence of the primary endpoint was observed (11.6% vs. 15.2% for those with high-intensity statin monotherapy; HR: 0.75; 95% CI: 0.70–0.79; *p* < 0.001). Compared with high-intensity statin monotherapy, with combination LLT, fewer statin discontinuations occurred (6.5% vs. 7.6%; HR: 0.85; 95% CI: 0.78–0.94: *p* < 0.001), and there was a lower occurrence of new-onset diabetes requiring medications (7.7% vs. 9.6%; HR: 0.80; 95% CI: 0.72–0.88; *p* < 0.001) [97,98,99].

Presently, combination LLT with high-intensity statins (rosuvastatin 20 mg or 40 mg; atorvastatin 40 mg and 80 mg) and ezetimibe (10 mg) are increasing. LLT including a combination of high–moderate-intensity statins and ezetimibe achieves better LDL-C management in CAD patients, and it has been associated with a significant improvement in patient compliance [100,101,102,103,104,105,106,107,108].

However, some data showed that statins in monotherapy continue to be the more prescribed treatment compared with combination therapies, despite guideline recommendations [47].

#### 3.1.2. Proprotein Convertase Subtilisin/Kexin Type 9 Inhibitors

PCSK9-i evolocumab and alirocumab are monoclonal antibodies that target PCSK9 in the liver. PCSK9 promotes the degradation of LDL-R on the hepatic cell surface. By inhibiting PCSK9, these agents increase the availability of LDL-R, thereby enhancing LDL-C clearance and significantly reducing circulating LDL-C levels [109,110,111,112] [Table 1].

In the FOURIER (Further Cardiovascular Outcomes Research With PCSK9 Inhibition in Subjects With Elevated Risk) trial, 27,564 patients with ASCVD were enrolled. The study demonstrated that adding evolocumab to statin therapy led to a significant reduction in the relative risk of the primary endpoint (a composite of CV death, MI, stroke, hospitalization for unstable angina, or coronary revascularization) (1344 patients 9.8% vs. 1563 patients 11.3%; HR, 0.85; 95% CI, 0.79 to 0.92; *p* < 0.001) [35]. Moreover, evolocumab was shown to significantly reduce the risk in patients at a high risk for MACEs (i.e., patients closer to the most recent MI, with multiple prior MIs, or with residual multivessel CAD) leading to lower LDL-C levels [118]. In a subanalysis of the FOURIER (Further Cardiovascular Outcomes Research With PCSK9 Inhibition in Subjects With Elevated Risk) trial, the authors highlighted that patients who achieved the lowest LDL-C cholesterol levels under evolocumab treatment experienced the lowest risk of MACEs, with no safety concerns observed even at VLDL levels (as low as 0.2 mmol/L) over a median follow-up of 2.2 years [117]. The FOURIER-OLE (Further Cardiovascular Outcomes Research With PCSK9 Inhibition in Subjects With Elevated Risk Open-Label Extension) study demonstrated that early and long-term LDL-C lowering with evolocumab resulted in further reductions in CV events compared with delayed treatment initiation. Additionally, the rate of adverse events remained consistently low over more than 8 years of follow-up [122].

A study testing early initiation of evolocumab in older patients (≥75 years) demonstrated that older patients with ASCVD derived cardiovascular benefits from treatment that were at least comparable to those observed in younger patients (HR: 0.79; 95% CI: 0.64–0.97). Notably, the number needed to treat to reduce the composite endpoint was more favorable in older patients. No significant adverse events were observed, supporting the safety of treatment across age groups. These findings may help inform future clinical recommendations [123,130].

The ODYSSEY Outcomes (Evaluation of Cardiovascular Outcomes After an Acute Coronary Syndrome During Treatment With Alirocumab) trial evaluated alirocumab in patients with a recent ACS (in the previous year) in addition to statin therapy in comparison to placebo in addition to statin. In this trial, 18,924 participants were enrolled, with the findings demonstrating that the risk of recurrent ischemic CV was lower in the alirocumab group than in the placebo group (HR, 0.85; 95% CI, 0.78 to 0.93; *p* < 0.001) [36].

Notably, the treatment with PCSK9-i demonstrated favorable changes in CAD in terms of stabilization and regression of atherosclerotic plaques with the support of invasive imaging, using both IVUS and Optical Coherence Tomography (OCT) [113,115,121].

According to the 2019 ESC guidelines, PCSK9-i introduction in lipid-lowering therapy in patients post ACS should be stepwise. This means that in patients naïve to LLT, PCSK9-i should be introduced if the LDL-C target is not achieved after a second evaluation of lipid levels after 4–6 weeks on the maximum tolerated dose of statin and ezetimibe combined. In addition, if patients are already on the maximum tolerated dose of statin and ezetimibe without achieving the LDL-C target, PCSK9-i can be started earlier with a class I recommendation and level A evidence [12]. The most recent 2025 focused update of the ESC guidelines stresses the concept of initiating the combination therapy early during ACS hospitalization in order to strike early and strongly impact LDL-C levels [3].

The degree of LDL-C reduction achieved with various LLT is well established. Moderate-intensity statins reduce LDL by approximately 30%, while high-intensity statins achieve about a 50% reduction. Adding ezetimibe to a high-intensity statin increases the reduction to around 65%. PCSK9-i alone lowers LDL-C by about 60%, which increases to approximately 75% when combined with a statin, and up to 85% when combined with both a statin and ezetimibe [58]. This suggests that an optimal combination therapy should be considered upfront, even in patients naïve to LLT, to achieve LDL-C targets as early as possible. The fast-track approach could be sustained by the efficacious and safe results already demonstrated in the EVOPACS (Evolocumab for Early Reduction of LDL-C Levels in Patients With Acute Coronary Syndromes) trial, where evolocumab was administrated in-hospital early after ACS and after 4 weeks in addition to statin therapy with 40 mg of atorvastatin in most patients (about 80% of participants), who had not been on previous statin treatment (0.79 mmol/L vs. 2.06 mmol/L; −40.7%, CI 95%, 45.2–36.2; *p* < 0.001)] [119]. Similar results were observed in the EVACS (Evolocumab in Acute Coronary Syndrome) study where evolocumab was administered early in patients with NSTEMI and in EPIC-STEMI (The Effects of Acute, Rapid Lowering of Low Density Lipoprotein Cholesterol with Alirocumab in Patients with ST Segment Elevation Myocardial Infarction Undergoing Primary PCI), a randomized trial enrolling patients with STEMI who received early alirocumab administration (35.9 ± 24 vs. 64.5 ± 27; *p* < 0.01) [114,120]. There is a need to define an ideal LDL-C threshold to initiate a fast-track treatment approach. According to a consensus document from the Italian Society of Interventional Cardiology (SICI-GISE), this cut off could be LDL-C >140 mg/dL [131]. The AT-TARGET-IT (Efficacy, safety, adherence and persistence of PCSK9-I in clinical practice: a single country, multicenter, observational study) registry is an investigator-initiated, multicenter, single-country study evaluating the use of PCSK9-i in routine clinical practice in Italy. It included 771 patients hospitalized for ACS who received early administration of a PCSK9-i, either during hospitalization or at discharge, to manage lipid levels [132]. This reflects an intensive and early LLT strategy, consistent with the “strike early–strike strong” approach [133]. The strike early–strike strong approach proved to be safe and effective, achieving the LDL-C target recommended by the ESC and American College of Cardiology/American Heart Association (ACC/AHA) guidelines in the majority of patients [12,132,134]. Reduction in residual CV risk in clinical practice was observed [132]. The strategy of early administration of PCSK9-i after an MI event has quickly become an intensive field of research and there are two ongoing trials aiming to investigate the efficacy of evolocumab in this clinical scenario: the EVOLVE-MI (EVOLocumab Very Early After Myocardial Infarction) trial (NCT05284747) and the AMUNDSEN (Evolocumab or Normal Strategies to Reach LDL-C Objectives in Acute Myocardial Infarction Upbound to PCI) trials (NCT04951856).

Furthermore, PCSK9-i is safe and effective, even as an alternative to statins in patients with statin intolerance. In the GAUSS-3 (Goal Achievement After Utilizing an Anti-PCSK9 Antibody in Statin Intolerant Subjects-3) trial, 511 adult patients with LDL-C not at target and a history of intolerance (muscle-related) to ≥2 or statins were enrolled and randomized to PCSK9-i or ezetimibe. Among patients with muscle-related statin intolerance, therapy with evolocumab resulted in a significantly greater reduction in LDL-C levels compared with ezetimibe after 24 weeks—at 24 weeks, ezetimibe vs. evolocumab: −16.7% (95% CI, −20.8% to −12.5%); absolute change, −31.2 mg/dL vs. −52.8% (95% CI, −55.8% to −49.8%) [116]. Additional beneficial effects of PCSK9-i may include their ability to reduce Lp(a) levels and exert anti-inflammatory actions.

Enlicitide decanoate is an oral, small-molecule peptide inhibitor of PCSK9, able to decrease LDL-C through a mechanism similar to that of monoclonal antibodies against PCSK9. In the recent phase 3 CORALreef HeFH randomized trial, the statin-added treatment with enlicitide was well tolerated and significantly reduced levels of LDL-C, apolipoprotein B, non-HDL-C, and lipoprotein(a) in 303 adult patients with HeFH [124].

Inclisiran is a small interfering ribonucleic acid that inhibits hepatic synthesis of PCSK9. Inclisiran lowers LDL-C levels considerably [126]. Inclisiran efficacy and safety has been analyzed in the ORION (A Study of Inclisiran in Participants With Homozygous Familial Hypercholesterolemia) program [125]. ORION-1 (Trial to Evaluate the Effect of ALN-PCSSC Treatment on Low Density Lipoprotein Cholesterol) A was the first trial in the ORION program to demonstrate that inclisiran significantly reduced PCSK9 and LDL-C levels in patients with a high CV risk with elevated baseline LDL-C, achieving a sustained lipid-lowering effect over time (−27.9% vs. −41.9%; −35.5% vs. −52.6%; *p* < 0.001) [127,128,135,136]. In ORION-10 (Inclisiran for Participants With Atherosclerotic Cardiovascular Disease and Elevated Low-density Lipoprotein Cholesterol) and ORION-11 (Inclisiran for Subjects with ASCVD or ASCVD-Risk Equivalents and Elevated Low-density Lipoprotein Cholesterol) trials, 1561 and 1617 patients, respectively, were randomly assigned to a placebo group or an inclisiran group, with inclisiran administered subcutaneously at a dose of 284 mg at day 1, day 90, and every 6 months on top of statin therapy. A reduction in LDL-C levels of approximately 50% was achieved with inclisiran in both ORION-10 and ORION-11 cohorts [126]. Inclisiran reduced LDL-C levels by approximately 50%, even in Japanese patients [137]. More injection-site adverse events occurred with inclisiran than with placebo [126]. Results were confirmed by a pooled analysis of seven clinical trials [138]. ORION-4 (A Randomized Trial Assessing the Effects of Inclisiran on Clinical Outcomes Among People With Cardiovascular Disease) (NCT03705234), which aims to evaluate CV outcomes in patients who already had a cardiac or cerebral vascular accident or who were already exposed to obstructive artery treatment, and VICTORION-2 PREVENT (Study of Inclisiran to Prevent Cardiovascular (CV) Events in Participants With Established Cardiovascular Disease) (NCT05030428), which aims to evaluate MACEs in patients with established ASCVD who are treated with inclisiran versus placebo therapy in adjunction to statin therapy, will provide further information on the protective effects of inclisiran in high-risk patients [127].

Of note, on the basis of the ORION-2 (A Study of ALN-PCSSC in Participants With Homozygous Familial Hypercholesterolemia) pilot study and the promising results of ORION-9 (Trial to Evaluate the Effect of Inclisiran Treatment on LDL-C in Subjects With HeFH) regarding the efficacy of inclisiran in Homozygous Familial Hypercholesterolemia (HoFH) and HeFH, ORION-5 (Study of Inclisiran in Participants With Homozygous Familial Hypercholesterolemia) was conducted, but inclisiran was not shown to significantly reduce LDL-C in HoFH, despite the great reduction in PCSK9 and the safety profile [139,140,141].

A recent report on the 1-year (part 1) results of the double-blind, placebo-controlled phase 3 study ORION-13 (Study to Evaluate Efficacy and Safety of Inclisiran in Adolescents With Homozygous Familial Hypercholesterolemia) in adolescents with HoFH showed that inclisiran was effective in lowering LDL-C in adolescents with HoFH and was well tolerated, suggesting that it could be a potentially useful addition to the treatment of adolescents with HoFH and achieving a minimum of LDLR residual activity [129].

#### 3.1.3. Bempedoic Acid

Bempedoic acid is a first-in-class ATP citrate lyase inhibitor. It acts on the cholesterol synthesis pathway, inhibiting cholesterol synthesis, and was demonstrated to lower LDL-C by approximately 23% in monotherapy, 18% when administered as a background of statin therapy, and 38% when combined with ezetimibe [142,143] [Table 2]. Indeed, a study evaluated the efficacy of 180 mg of oral bempedoic acid in combination with ezetimibe in patients with ASCVD and hypercholesterolemia on maximally tolerated statin therapy. The study demonstrated that the fixed-dose combination significantly reduced LDL-C levels compared to placebo or individual oral monotherapies and was well tolerated, showing a favorable safety profile in this high-CV-risk population [142]. Furthermore, in the CLEAR (Cholesterol Lowering via Bempedoic Acid, an ACL-Inhibiting Regimen) Outcomes trial, 13,970 statin-intolerant patients were randomly assigned to receive bempedoic acid or placebo in addition to the maximum tolerated statin dose. Bempedoic acid treatment was associated with a lower rate of MACEs and a higher rate of gout and cholelithiasis (819 patients (11.7%) vs. 927 (13.3%); HR, 0.87; 95% CI, 0.79 to 0.96; *p* = 0.004) [144]. After 58 weeks of treatment, bempedoic acid added to statin therapy was demonstrated to significantly reduce LDL-C in patients with ASCVD, with no observations of a higher rate of overall adverse events with respect to placebo [143]. Consistent results have been observed in a population at a high risk of CV in the CLEAR Wisdom (Effect of Bempedoic Acid vs. Placebo Added to Maximally Tolerated Statins on Low-Density Lipoprotein Cholesterol in Patients at High Risk for Cardiovascular Disease) trial [145]. Ongoing randomized clinical trials evaluating early intensive LLT with statin plus ezetimibe plus bempedoic acid will provide further evidence in patients with ACS (ES-BempeDACS; EudraCT 2021-006550-31) [146]. According to the most recent 2025 ESC focused update on dyslipidemias, bempedoic acid is recommended in patients that cannot take statin, to achieve LDL-C goals with a class I recommendation and level B evidence [3]. Moreover, the addition of bempedoic acid to the maximally tolerated dose of a statin in association or not with ezetimibe should be considered in patients at a high or very high risk, to achieve the LDL-C target with a class IIa recommendation and level C evidence [3,4].

#### 3.1.4. Obicetrapib

Obicetrapib is a new LLT drug that inhibits Cholesteryl Ester Transfer Protein (CETP). In the BROADWAY (The Randomized Study to Evaluate the Effect of Obicetrapib on Top of Maximum Tolerated Lipid-Modifying Therapies) trial, in patients with ASCVD or HeFH receiving maximum tolerated doses of LLT, it reduced LDL-C levels by almost 30% (obicetrapib group: LDL-C cholesterol level was −29.9%; 95% CI, −32.1 to −27.8), as compared with the placebo group (2.7%; 95% CI, −0.4 to 5.8), resulting in a between-group difference of −32.6 percentage points (95% CI, −35.8 to −29.5; *p* < 0.001) [147]. Moreover, in the TANDEM (Fixed-dose combination of obicetrapib and ezetimibe for LDL-C reduction) trial, fixed-dose combination (FDC) therapy of obicetrapib and ezetimibe in a single-pill formulation significantly reduced LDL-C in patients with pre-existing ASCVD or at a high risk for ASCVD (LDL-C reduction with FDC −48.6%; 95% CI −58.3 to −38.9 versus placebo, −27.9%; −37.5 to −18.4 versus ezetimibe, and −16.8%; −26.4 to −7.1 versus obicetrapib [148]. Intriguingly, obicetrapib was demonstrated to reduce about 57% of Lp(a) levels in the ROSE trial (Randomized Study of Obicetrapib as an Adjunct to Statin Therapy) [149,150]. More data are needed.

#### 3.1.5. Omega-3 Fatty Acids and Icosapent Ethyl

Eicosapentaenoic acid (EPA), a member of the omega-3 fatty acid family, is used at pharmacological doses of 2–4 g/day to lower TG levels. It primarily affects serum lipid and lipoprotein concentrations, particularly by reducing VLDL levels. Its mechanism of action is probably linked to the interaction with Peroxisome Proliferator-Activated Receptors (PPARs) and consequently reduction in the secretion of ApoB [16] [Table 3]. Moreover, it seems it has a pleiotropic effect with anti-inflammatory action, modulating the atherosclerotic process [151].

The efficacy of omega-3 fatty acids in lowering TG serum levels has been reported in different clinical studies: ANCHOR (Efficacy and Safety of Eicosapentaenoic Acid Ethyl Ester Therapy in Statin-Treated Patients with Persistent High Triglycerides), MARINE (Multi-Center, plAcebo-Controlled, Randomized, Double-blINd, 12-Week Study with an Open-Label Extension), EVOLVE (The EpanoVa fOr Lowering Very High triglyceridEs), EVOLVE II [159,160,161,162].

Despite the promising findings from the JELIS (Effects of eicosapentaenoic acid on major coronary events in hypercholestero laemic patients) trial (using 1.8 g/day of EPA) and the study by Nosaka et al. [153] (also using 1.8 g/day of EPA), which demonstrated a reduction in MACEs among patients with established CAD and post-ACS, the STRENGTH (Effect of High-Dose Omega-3 Fatty Acids vs. Corn Oil on Major Adverse Cardiovascular Events in Patients at High Cardiovascular Risk) trial, conducted in 13,078 patients with high CV risk and hypertriglyceridemia, showed no significant difference in outcomes between high-dose EPA (4 g/day) and a corn oil placebo (12% vs. 12.2%; HR, 0.99; CI 95%, 0.90–1.09; *p* = 0.84). These results do not support the routine use of EPA in patients at high risk of CV [152,153,154].

The OMEMI (Omega-3 Fatty acids in Elderly with Myocardial Infarction) trial is a randomized clinical trial that added 1.8 g of n-3 Polyunsaturated Fatty Acids (PUFAs), 930 mg EPA, and 660 mg docosohexaenoic acid versus placebo daily to the standard of care in patients from 70 to 82 years of age who experimented a recent (2–8 weeks) MI. A significant reduction in MACEs in patients treated with 1.8 g n-3 PUFA daily for 2 years was not observed (21.4% vs. 20%; HR, 1.08; CI 95%, 0.82–1.41, *p* = 0.60) [156,157]. These results are consistent with the previous OMEGA (Effect of Omega-3 fatty acids on the reduction in sudden cardiac death after MI) trial and those derived from another study, both conducted in patients after MI [163]. Results of meta-analyses are discordant regarding the CV benefits of EPA/PUFAs [164,165,166,167].

Icosapent ethyl (IPE) is a highly purified and stable EPA ethyl ester that has been shown to lower TG levels [159,160].

In fact, the REDUCE-IT (Cardiovascular Risk Reduction with Icosapent Ethyl for Hypertriglyceridemia) study evaluated the benefits of Icosapent Ethyl (IPE) on ASCVD outcomes in more than 8000 patients with elevated levels of TG, demonstrating that high doses (at least 2 g/Day) of IPE reduced about 25% of the relative risk of MACEs compared to placebo (17.2% vs. 22%; HR, 0.75; CI 95%, 0.68–0.83; *p* < 0.001) [158].

The RESPECT-EPA (Randomized Trial for Evaluation in Secondary Prevention Efficacy of Combination Therapy—Statin and Eicosapentaenoic Acid) trial assessed the clinical benefits of 1.8 g/day of IPE and statin treatment in patients with CAD. A numerical reduction in CV events in the IPE group was observed, even if not statistically significant [9.1% vs. 12.6%; HR, 0.79; CI 95%, 0.62–1.00; *p* = 0.055) [155].

There is a clear discordance between data. This could be related to several reasons, and probably the most relevant regards the different doses of omega-3 used among the trials. It seems that the omega-3 fatty acids were administered at the same dose used in previous trials (above 1 g/day), which appears to be insufficient to affect plasma lipids, as the dose required to decrease plasma TG is >2 g/day [16]. IPE is the only member of the omega-3 fatty acid family that has been shown to lower TG levels [159,160].

#### 3.1.6. Lipoprotein(a)

Lipoprotein(a) [Lp(a)] is an LDL-C particle with an Apo(a) protein covalently bound to its ApoB component [54]. There are currently no approved drugs that have demonstrated a reduction in CV events related to lowering blood Lp(a) levels. Among the available medications, PCKS9-i has been shown to reduce Lp(a) levels by approximately 20–30% [168,169,170].

Olpasiran is a small interfering RNA presently under investigation that has been demonstrated to reduce Lp(a) synthesis in the liver. The OCEAN(a)-DOSE TRIAL (Small Interfering RNA to Reduce Lp(a) in Cardiovascular Disease) enrolled 281 ASCVD patients randomly assigned to placebo or olpasiran and showed that olpasiran significantly reduced Lp(a) concentrations. Doses of 10 mg, 75 mg, and 225 were investigated. At 36 weeks, the mean percent change in the Lp(a) concentration in the placebo group was an increase of 3.6% (95% confidence interval [CI], −0.1 to 7.3), while there were significant reductions in the Lp(a) concentration in all olpasiran groups. Specifically, the placebo-adjusted mean percent change in the Lp(a) levels was −70.5% (95% CI, −75.1 to −65.9) for the 10 mg dose every 12 weeks, −97.4% (95%, CI, −102.0 to −92.8) for the 75 mg dose every 12 weeks, −101.1% (95% CI, −105.8 to −96.5) for the 225 mg dose every 12 weeks, and −100.5% (95% CI, −105.2 to −95.8) for the 225 mg dose every 24 weeks (*p* < 0.001 for all comparisons with baseline). At 48 weeks follow-up, the placebo-adjusted mean percent change in the Lp(a) concentration with olpasiran was −68.5% (95% CI, −74.3 to −62.7) for the 10 mg dose every 12 weeks, −96.1% (95% CI, −101.9 to −90.3) for the 75 mg dose every 12 weeks, −100.9% (95% CI, −106.7 to −95.0) for the 225 mg dose every 12 weeks, and −85.9% (95% CI, −91.8 to −80.1) for the 225 mg dose every 24 weeks [171].

Another approach under investigation is the selective reduction in Lp(a) concentrations. RNA-based therapies, including antisense oligonucleotides, are currently being evaluated in clinical settings. In particular, the hepatocyte-directed antisense oligonucleotide pelacarsen was demonstrated to reduce Lp(a) levels in a dose-dependent manner in patients with elevated Lp(a) levels and established ASCVD. Compared with placebo, pelacarsen resulted in dose-dependent decreases in Lp(a) (2% vs. −29% to −67%; *p* = 0.001-<0.0001) [172,173]. Studies in patients with both normal and elevated Lp(a) levels have shown reductions greater than 90% [174].

Obicetrapib, a CETP inhibitor, was demonstrated to reduce Lp(a) levels of about 57% [149,150].

These strategies have been tested in different trials, and an outcome trial is planned to assess whether Lp(a) lowering translates into a reduction in CV risk [172,175,176,177,178].

### 3.2. Targeting Inflammation

#### 3.2.1. Colchicine

**Figure 3:**
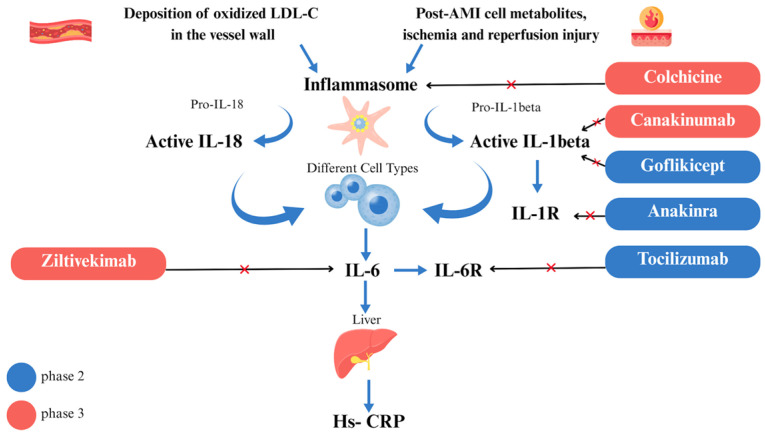
Focus on the mechanism of action of anti-inflammatory drugs. Colchicine disrupts tubulin polymerization, thereby interfering with multiple inflammatory pathways and components of innate immunity. In particular, colchicine inhibits neutrophil activity and cytokine release, including IL-1 and IL-6, inhibiting the inflammasome. Canakinumab is a fully human monoclonal antibody that inhibits IL-1ꞵ. Goflikicept (RPH-104) is a hybrid protein that selectively binds and inactivates circulating IL-1ß and circulating IL-1α. Anakinra is an IL-1R antagonist. Ziltivekimab is a human monoclonal antibody that targets the IL-6 ligand. Colchicine, canakinumab, and ziltivekimab have been tested in phase 3 trials; goflikicept, anakinra, and tocilizumab have been tested in phase 2 trials. Inflammation plays a crucial role from the early formation of atherosclerotic plaque until its destabilization; these drugs contribute to the reduction in hs-CRP levels, thus reducing the residual inflammatory risk in patients with ASCVD. AMI: Acute Myocardial Infarction; ASCVD: Atherosclerotic Cardiovascular Disease; Hs-CRP: High-sensitivity C-Reactive Protein; IL: Interleukin; LDL-C: Low-Density Lipoprotein Cholesterol. X indicates inhibition by the drug.

Colchicine is a drug known since ancient times. It is extracted from *Colchinum autumnale*, and in ancient Egypt, it was used as an antirheumatic and antigout plant-based remedy [179,180]. Today, in cardiology, it is commonly used to treat acute and chronic pericardial diseases, given its anti-inflammatory properties (Figure 3) [181,182,183].

The anti-inflammatory role of colchicine is related to its effects on tubulin. Colchicine disrupts tubulin polymerization, thereby interfering with multiple inflammatory pathways and components of innate immunity. In particular, colchicine inhibits neutrophil activity and cytokine release, including IL-1 and IL-6 [179,180].

In a pilot study, colchicine was demonstrated to reduce infarct size in STEMI patients, suggesting a potential beneficial role of colchicine after PCI [184]. The first studies trying to demonstrate the beneficial effects of colchicine in ACS after PCI were not sufficiently powered and needed further evidence [185,186]. The COPS (Colchicine in Patients With Acute Coronary Syndrome) trial pointed out that colchicine in addiction to standard of care did not significantly reduce CV events compared to placebo in patients with ACS. A higher rate of non-CV death was observed [187]. In the COVERT-MI (Colchicine for Left Ventricular Infarct Size Treatment in Acute Myocardial Infarction) trial, the acute effects of colchicine in 192 patients with STEMI undergoing cardiac magnetic resonance imaging were investigated [188]. At 5 days and again at 3 months, no significant differences were observed between colchicine and placebo in terms of infarct size (IS) or left ventricular remodeling.

COLCOT (Colchicine CV outcomes) was a larger trial conducted in 4745 patients randomized to colchicine or placebo groups within 30 days after myocardial infarction, to receive colchicine at a dose of 0.5 mg/die orally or placebo. Colchicine treatment demonstrated a 23% relative reduction in ischemic cardiovascular events in comparison to placebo, although a major incidence of pneumonia was observed in patients administered colchicine in comparison to placebo [189] [Table 4].

In stable CAD, colchicine was also demonstrated to reduce the risk of cardiovascular events [194] [Table 5]. In the randomized, controlled, double-blind LoDoCo2 (Low Dose Colchicine) trial, involving 5522 patients with CCS, the risk of cardiovascular events was significantly lower among those who received 0.5 mg of colchicine once daily than those who received placebo (incidence, 2.5 vs. 3.6 events per 100 person-years; HR, 0.69; 95% CI, 0.57 to 0.83; *p* < 0.001) [195].

Interestingly, considering the known vascular inflammation status that occurs after PCI, in the COLCHINE-PCI (Effects of Acute Colchicine Administration Prior to Percutaneous Coronary Intervention) trial, administration of a high dose of colchicine (1.8 mg) before PCI was demonstrated to significantly reduce inflammation markers (57.3% vs. 64.2; *p* = 0.19). Nevertheless, the trial did not show a reduction in the risk of PCI-related myocardial injury compared to placebo [191].

In the recent CLEAR (Cholesterol Lowering via Bempedoic Acid, an ACL-Inhibiting Regimen) trial, a multicenter, randomized trial, 7062 patients with a recent MI were randomly assigned to receive colchicine or placebo or spironolactone or placebo. The primary efficacy outcome was a composite of death from CV causes, MI, stroke, or unplanned ischemia-driven coronary revascularization. The drug was administered soon after the ACS and continued for a median of three years. Colchicine did not significantly reduce the primary outcome (HR, 0.99; 95% CI, 0.85 to 1.16; *p* = 0.93). A higher incidence of diarrhea was observed with colchicine compared to placebo, consistent with findings from previous studies. Additionally, a reduction in CRP levels was noted (95% CI, −1.81 to −0.75) [193].

A recent meta-analysis demonstrated the potential benefit of colchicine in secondary prevention [196]. Nine trials were included, with a total of 30,695 patients with known CAD or stroke (15,255 patients treated with colchicine versus 15,404 without). Compared with patients who did not receive colchicine, patients administered colchicine had a relative risk (RR) of 0.88 [95% CI 0.81–0.95, *p* = 0.002] for the primary composite outcome, including an RR of 0.94 for CV death (95% CI 0.78–1.13, *p* = 0.5), an RR of 0.84 for MI (95% CI 0.73–0.97, *p* = 0.016), and an RR of 0.90 for stroke (95% CI 0.80–1.02, *p* = 0.09). Colchicine was associated with an RR of 1.35 for hospitalization for gastrointestinal events (95% CI 1.10–1.66, *p* = 0.004) with no increase in hospitalization for pneumonia, newly diagnosed cancers, or non-CV death. It was demonstrated that colchicine reduced the composite of CV death, MI, or stroke by 12% in patients with prior CAD or stroke [196].

The effects of colchicine on atherosclerotic plaque using computerized tomography evaluation have been analyzed. In an observational trial involving 80 patients with ACS within the previous 30 days, it was found that adding 0.5 mg of colchicine to OMT improved the morphology of potentially vulnerable atherosclerotic plaques. This benefit may be attributed to colchicine’s anti-inflammatory action on plaque components [190]. COLOCT (Colchicine Cardiovascular Outcomes Trial) was a prospective, single-center, randomized, double-blind clinical trial analyzing the morphology of plaque at OCT in patients affected by ACS at the moment of acute event and at 12 months. In total, 128 patients with ACS with OCT evidence of a lipid-rich plaque (lipid pool arc >90°) were included. They were randomly assigned to receive colchicine at a dosage of 0.5 mg once daily or a placebo for 12 months. The primary endpoint evaluated the change in minimal fibrous cap thickness. Colchicine significantly improved plaque stability with the inhibition of vascular inflammation (51.9 µm; 95% CI, 32.8–71 vs. 87.2 µm; CI 95%, 69.9–104.5). This suggests colchicine could be considered as an addition to medical therapy in patients in secondary prevention [192]. Nevertheless, the previously described studies based on imaging were conducted on a small sample size. Further data are needed to confirm the effectiveness and security of colchicine in ACS patients.

The most recent ESC guidelines recommend that low-dose colchicine at 0.5 mg daily should be considered to reduce the risk of MI, stroke, and need for revascularization in secondary prevention for patients with atherosclerotic CAD, with a class IIa recommendation and level A evidence [4]. However, these guidelines were released prior to the CLEAR (Cholesterol Lowering via Bempedoic Acid, an ACL-Inhibiting Regimen) trial results.

There are ongoing trials that are expected to advance our current knowledge regarding the role of colchicine in ACS: the large-scale TACTIC (Treatment of ACuTe Coronary Syndromes With Low-dose colchicine, NCT06215989) trial, COL-BE PCI (Colchicine in Belgium in Patients With Coronary Artery Disease, NCT06095765), and COLCARDIO-ACS (Colchicine Cardiovascular Outcomes in Acute Coronary Syndrome Study, ACTRN12616000400460).

#### 3.2.2. Methotrexate

Methotrexate (MTX) is an antimetabolite commonly used for the management of auto-immune illness, such as rheumatoid arthritis, chronic bowel disease, psoriasis, and cancer. Moreover, it can be used in patients subjected to organ transplantation due to its strong immunomodulatory activity [197].

According to its use, MTX performs a different mechanism of action. When used for autoimmune disease, it inhibits the AICAR enzyme, disrupting the metabolism of adenosine and guanine. Accumulation of adenosine causes a reduction in T- and B-cell activity, thus exerting an anti-inflammatory effect [198].

Observational studies have observed that use of a low dose of MTX in patients affected by rheumatoid arthritis or psoriatic rheumatoid arthritis had fewer CV events in comparison to patients who received other therapies or placebo [199,200].

Considering the marked anti-inflammatory activity of MTX, a role for MTX in atherosclerosis was hypothesized. Ferrari and colleagues have suggested that a formulation of MTX incorporated into a lipid nano-emulsion (LDE-MTX) could be beneficial and safe [201]. In this small study, 32 patients after a first anterior STEMI were randomized to receive LDE-MTX (40 mg/m^2^ intravenous infusion) or LDE-placebo for 6 weeks. At 90 days, there was no difference in left ventricle end-diastolic volume (LVEDV) between the groups. The CIRT trial was a double-blind trial in which 4786 patients with previous ACS or multivessel CAD were randomized to low-dose MTX or matching placebo. Patients were additionally affected by T2DM or metabolic syndrome. In patients with stable CAD, a low dose of MTX did not reduce CV events nor did it significantly reduce IL-1ꞵ, IL-6, or CRP in comparison to placebo. Moreover, patients treated with MTX experienced a higher frequency of cell basal skin cancer than those who were administered a placebo. This is an unexpected effect that needs further investigation [202]. The authors explain the difference between the results of the CIRT (Cardiovascular Inflammation Reduction Trial) and the results of the CANTOS trial, considering the populations examined. Indeed, in CIRT (Cardiovascular Inflammation Reduction Trial), patients with stable CAD and T2DM or metabolic syndrome independent from CRP levels at baseline were included, while in the CANTOS (Anti-inflammatory Therapy with Canakinumab for Atherosclerotic Disease) trial, patients with elevated residual inflammatory risk with persistently high levels of hsCRP (≥2 mg/L) were included [38]. This could explain the difference between the results of the two studies. In the THETYS (The Effects of mETHotrexate Therapy on ST Segment Elevation MYocardial InfarctionS) trial, 84 patients with STEMI were randomly assigned to receive either MTX or placebo. There was no significant reduction in infarct size; rather, LVEF was lower, and there was a higher median serum glutamic–pyruvic transaminase level in patients who received MTX [203].

MTX has been shown to reduce CRP levels in inflammatory conditions characterized by elevated CRP, such as rheumatoid arthritis [204] [Table 6].

#### 3.2.3. Canakinumab

IL-1ꞵ is an inflammatory interleukin involved in atheroma development and growth. Furthermore, IL-1ꞵ activates the IL-6 signaling pathway [66]. Canakinumab is a fully human monoclonal antibody that targets IL-1ꞵ, and it has already been approved as a medical treatment in rheumatological disorders [208,209,210]. Ridker and colleagues showed the anti-inflammatory effect of canakinumab in a phase 2 randomized placebo-controlled trial on diabetic patients with a high CV risk. Canakinumab significantly reduced IL-6 and CRP. No lipid reduction was observed [205]. Considering these results, Ridker and colleagues tested its potential as an anti-inflammatory therapy in the CANTOS (Anti-inflammatory Therapy with Canakinumab for Atherosclerotic Disease) trial. CANTOS (Anti-inflammatory Therapy with Canakinumab for Atherosclerotic Disease) was a randomized, double-blind trial on canakinumab involving 10,061 patients with previous MI and high plasma levels of hsCRP (≥2 mg/L) despite the use of concomitant medical therapy for secondary prevention. The CANTOS (Anti-inflammatory Therapy with Canakinumab for Atherosclerotic Disease) trial compared three doses of canakinumab, 50 mg, 150 mg, and 300 mg, with placebo. All treatments were administered subcutaneously once every three months. The primary endpoint included nonfatal MI, nonfatal stroke, or CV death. Only the 150 mg dose of canakinumab met the primary endpoint, as well as the secondary endpoint, which included the components of the primary endpoint plus hospitalization for unstable angina requiring urgent revascularization. No significant LDL-C reduction was observed with any dose of the drug. A total of 150 mg of canakinumab administered subcutaneously every three months led to a significant reduction in CV events compared to the placebo, independent of LLT. Additionally, reductions in hsCRP and IL-6 levels were observed. However, treatment with canakinumab was associated with an increased risk of infections, including a significantly higher incidence of fatal infections and sepsis compared to placebo [38]. Intriguingly, an additional analysis of the CANTOS (Anti-inflammatory Therapy with Canakinumab for Atherosclerotic Disease) trial hypothesized the possibility that anti-inflammatory therapy targeting IL-1ꞵ with canakinumab could reduce lung cancer incidence and mortality [206]. In conclusion, the CANTOS (Anti-inflammatory Therapy with Canakinumab for Atherosclerotic Disease) trial directly tested the hypothesis of inflammation in atherosclerosis, demonstrating a possible beneficial effect of reduction in inflammatory levels in patients at a high risk of CV events. Nonetheless, more evidence is needed to find the right drug with the best balance between beneficial effect and adverse events [Table 6].

#### 3.2.4. Ziltivekimab, Anakinra, Goflikicept

Ziltivekimab is a human monoclonal antibody that targets the IL-6 ligand. The RESCUE trial was a double-blind, placebo-controlled, phase II study conducted in patients with chronic kidney disease and a high risk of CV. Available data from the trial support the finding that monthly administration of ziltivekimab reduces plasma levels of hsCRP more effectively than canakinumab [38]. Moreover, ziltivekimab reduces Lp(a) levels. Adverse events were not significant. Above all, no fatal infections or sepsis were recorded. Although promising, the RESCUE trial showed several limitations; it was interrupted earlier due to the COVID-19 pandemic, and the number of patients and months of follow-up were modest. Data on a larger sample and for a longer time is needed [207]. Based on the RESCUE (Trial to Evaluate Reduction in Inflammation in Patients With Advanced Chronic Renal Disease Utilizing Antibody Mediated IL-6 Inhibition) results, Ridker and colleagues are conducting a new trial, ARTEMIS (A Research Study to Look at How Ziltivekimab Works Compared to Placebo in People With a Heart Attack) (NCT06118281), to evaluate whether ziltivekimab can reduce recurrent cardiovascular events in patients with CAD. Specifically, ARTEMIS (A Research Study to Look at How Ziltivekimab Works Compared to Placebo in People With a Heart Attack) is an ongoing interventional, randomized, parallel-group, double-blind, placebo-controlled, multicenter, multi-national cardiovascular outcome trial on the effects of ziltivekimab compared to placebo in patients after ACS, added to the standard of care in approximately 10,000 patients. It is estimated to be completed in 2026. Patients administered ziltivekimab will receive a loading dose subcutaneously as soon as possible after ACS; then, they will receive a maintenance dose once monthly.

Other molecules are under investigation to reduce inflammation in patients in secondary prevention.

Phase II clinical trials, the VCUART trials (Virginia Commonwealth University Anakinra Response Trials), demonstrated that anakinra inhibits the acute inflammatory response and prevention of HF after STEMI [211,212,213,214,215].

Goflikicept (RPH-104) is a hybrid protein that selectively binds and inactivates circulating IL-1ß and circulating IL-1α [216]. In a recent phase II randomized trial, goflikicept at 80 mg or 160 mg reduced hs-CRP levels in patients with STEMI compared to placebo [217].

Even if promising results come from anti-interleukin drugs, more data are necessary to investigate their safety and efficacy.

## 4. Conclusions

Patients undergoing PCI remain at a very high risk for residual CV despite revascularization, highlighting the need for comprehensive secondary prevention strategies. Reducing the risk of MACEs requires early and effective pharmacological interventions, including aggressive lipid-lowering and anti-inflammatory agents, alongside lifestyle modifications. Specifically, LLT is strongly recommended, and LDL-C targets need to be increasingly monitored and reached through the use of high-dose potent statins and ezetimibe and eventually integrated in triple therapy with bempedoic acid or PCSK9-inhibitors based on the basal value, the clinical context, and the distance from the target. Also, triglyceride levels are associated with CV risk independently of LDL-C levels; thus, high-dose icosapent ethyl (2 × 2 g/day) should be considered in combination with a statin in high-risk or very-high-risk patients with elevated triglyceride levels (fasting triglyceride level 135–499 mg/dL) to reduce the risk of cardiovascular events. On the other hand, despite the fact that growing evidence supports the role of inflammation after PCI, from plaque progression and destabilization to the expansion of myocardial injury and adverse left ventricular remodeling, the current data on anti-inflammatory agents are still limited and conflicting. Colchicine represents the only anti-inflammatory agent recommended by guidelines, although the recent CLEAR trial has generated skepticism due to its findings contradicting previous evidence. Importantly, individualized risk assessment based on clinical profile, comorbidities, and biomarkers should guide the OMT to improve outcomes and mitigate adverse effects. Ongoing and future studies will assess some of these strategies, and these new important data will advance our current knowledge and could change our current recommendations and practice.

## Figures and Tables

**Table 1:** Main randomized trials of treatment with PCSK9-I in patients with CVD and high CV risk.

Trial, Year	Patients (*n*)	Population Characteristics	Randomized Arms	Primary Endpoint	Main Results	Conclusions
ODYSSEY OUTCOMES trial (2018) [113]	18,924	Patients who had ACS 1 to 12 months earlier, had LDL-C value ≥70 mg/dL, non-HDL ≥100 mg/dL or APOB ≥80 mg/dL, in HI statin therapy	To receive alirocumab or placebo every 2 weeks	A composite of death from coronary heart disease, nonfatal MI, fatal or nonfatal ischemic stroke, or unstable angina requiring hospitalization	A composite primary end-point event occurred in 903 patients (9.5%) in the alirocumab group and in 1052 patients (11.1%) in the placebo group (hazard ratio, 0.85; CI 95%, 0.78 to 0.93; *p* < 0.001)	The risk of recurrent ischemic CV events was lower among those who received alirocumab than among those who received placebo
EPIC STEMI trial (2022) [114]	68	Patients with STEMI undergoing primary PCI	To receive alirocumab plus HI statin or placebo plus HI statin	Percentage of LDL-C reduction up to 6 weeks	LDL-C decreased by 72.9% with alirocumab versus 48.1% with the sham control, for a mean between-group difference of −22.3% (*p* < 0.001)	Alirocumab reduced LDL-C by 22% compared with sham control on a background of HI statin therapy
PACMAN-AMI trial (2022) [115]	300	Patients with acute MI undergoing primary PCI	To receive alirocumab plus HI statin or placebo plus HI statin	The change in IVUS-derived percent atheroma volume	At 52 weeks, mean change in percent atheroma volume was −2.13% with alirocumab vs. −0.92% with placebo (difference, −1.21%; CI 95%, −1.78% to −0.65%; *p* < 0.001)	The addition of subcutaneous biweekly alirocumab, compared with placebo, to HI statin therapy resulted in significantly greater coronary plaque regression in non-infarct-related arteries after 52 weeks
GAUSS-3 trial (2016) [116]	511	Patients with muscle-related statin intolerance	Phase A: used a 24 week crossover procedure with atorvastatin or placebo to identify patients having symptoms only with atorvastatin but not placebo. In phase B, after a 2-week washout, patients were randomized to ezetimibe or evolocumab for 24 weeks	Coprimary end points were the mean percent change in LDL-C level from baseline to the mean of weeks 22 and 24 levels and from baseline to week 24 levels	For the mean of weeks 22 and 24, LDL-C level with ezetimibe was 183.0 mg/dL; mean percent LDL-C change, −16.7% (95% CI, −20.5% to −12.9%), absolute change, −31.0 mg/dL and with evolocumab was 103.6 mg/dL; mean percent change, −54.5% (95% CI, −57.2% to −51.8%); absolute change, −106.8 mg/dL (*p* < 0.001). LDL-C level at week 24 with ezetimibe was 181.5 mg/dL; mean percent change, −16.7% (95% CI, −20.8% to −12.5%); absolute change, −31.2 mg/dL and with evolocumab was 104.1 mg/dL; mean percent change, −52.8% (95% CI, −55.8% to −49.8%); absolute change, −102.9 mg/dL (*p*< 0.001)	Among patients with statin intolerance related to muscle-related adverse effects, the use of evolocumab compared with ezetimibe resulted in a significantly greater reduction in LDL-C levels after 24 weeks
FOURIER trial (2017) [117]	27,564	Patients with ASCVD and LDL-C levels ≥70 mg/dL who were receiving statin therapy	To receive evolocumab or placebo	A composite of CV death, MI, stroke, hospitalization for unstable angina, or coronary revascularization	Evolocumab treatment significantly reduced the risk of the primary end point (1344 patients [9.8%] vs. 1563 patients [11.3%]; HR, 0.85; CI 95%, 0.79 to 0.92; *p* < 0.001)	Inhibition of PCSK9 with evolocumab on a background of statin therapy lowered LDL-C levels to a median of 30 mg per deciliter (0.78 mmol per liter) and reduced the risk of CV events
FOURIER sub-analysis (2018) [118]	22,351	Patients with a prior MI (most recent MI, number of prior MIs, and presence of residual multivessel CAD)	To receive evolocumab or placebo	A composite of CV death, MI, stroke, hospitalization for unstable angina, or coronary revascularization	Reduction of the primary endpoint of 20% (HR, 0.80; 95% CI, 0.71–0.91), 18% (HR, 0.82; 95% CI, 0.72–0.93), and 21% (HR, 0.79; 95% CI, 0.69–0.91) for those with more recent MI, multiple prior MIs, and residual multivessel CAD	Patients closer to their most recent MI, with multiple prior MIs, or with residual multivessel CAD are at high risk for MACEs and experience substantial risk reductions with LDL-C lowering with evolocumab
EVOPACS trial (2019) [119]	308	Patients hospitalized for ACS with elevated LDL-C levels	To receive evolocumab or placebo	Percentage change in calculated LDL-C over 8 weeks	Mean LDL-C levels decreased from 3.61 to 0.79 mmol/l at week 8 in the evolocumab group, and from 3.42 to 2.06 mmol/l in the placebo group; the difference in mean percentage change from baseline was 40.7% (CI 95%: 45.2 to 36.2; *p* < 0.001)	Evolocumab added to HI statin therapy was well tolerated and resulted in substantial reduction in LDL-C levels, rendering >95% of patients within currently recommended target levels
EVACS trial (2020) [120]	57	Patients with NSTEMI and troponin I ≥5 ng/mL	To receive evolocumab or placebo	Change in LDL-C value (mg/dl) at hospital discharge and 30-day follow-up	LDL-C decreased from baseline by day 1 in the evolocumab group (70.4 ± 27 mg/dL; *p* < 0.01 versus baseline), and was lower than that in the placebo group by day 3 (*p* = 0.02 versus placebo). At 30-day follow-up LDL-C was 35.9 mg/dL ± 24 for evolocumab group vs. 64.5 mg/dL ± 27 for placebo (*p* < 0.01)	Evolocumab initiated in the hospital early after ACS rapidly and significantly reduces LDL-C in just 24 h
HUYGENS trial (2022) [121]	161	Patients with NSTEMI	To receive evolocumab or placebo	The change in the minimum fibrous cap thickness and maximum lipid arc throughout the imaged arterial segment	The evolocumab group demonstrated a greater increase in minimum fibrous cap thickness (+42.7 vs. +21.5 mm; *p* = 0.015) and decrease in maximum lipid arc (57.5° vs. 31.4°; *p* = 0.04) and macrophage index (3.17 vs 1.45 mm; *p* = 0.04) throughout the arterial segment	The combination of statin and evolocumab after a NSTEMI produces favorable changes in coronary atherosclerosis consistent with stabilization and regression
FOURIER-OLE trial (2022) [122]	6635	Patients completing FOURIER at participating sites were eligible to receive evolocumab in 2 open-label extension studies	To receive evolocumab or placebo	The primary end point was the incidence of adverse events	During the follow-up period, patients originally randomized in the FOURIER trial to evolocumab versus placebo had a 15% lower risk of CV death, MI, stroke or hospitalization for unstable angina or coronary revascularization (HR, 0.85 [CI 95%, 0.75–0.96]; *p* = 0.008); a 20% lower risk of CV death, MI or stroke (hazard ratio, 0.80 [CI 95%, 0.68–0.93]; *p* = 0.003); and a 23% lower risk of CV death (hazard ratio, 0.77 [95% CI, 0.60–0.99]; *p* = 0.04)	Long-term LDL-C lowering with evolocumab was associated with persistently low rates of adverse events for >8 years that did not exceed those observed in the original placebo arm during the parent study and led to further reductions in CV events compared with delayed treatment initiation
FOURIER Trial substudy (2025) [123]	27,564	Patients with ASCVD	To receive evolocumab or placebo	A composite of CV death, MI, stroke, hospitalization for unstable angina, or coronary revascularization stratified by age (<75 vs. >75)	The primary endpoint at least as well in older (HR: 0.79; CI 95%: 0.64–0.97) as in younger patients (HR: 0.86; 95% CI: 0.80–0.92; *p* interaction = 0.43). The absolute risk reductions were 5.4% (95% CI: −2.0% to 12.8%) in older and 2.3% (95% CI: 0.1–4.5%) in younger patients	Early initiation of long-term evolocumab provides older patients with ASCVD CV benefits at least as good as those observed in younger patients, with a more favorable number needed to treat in older patients for reducing a composite endpoint and no significant safety concerns
CORALreef HeFH trial (2025) [124]	303	Patients with HeFH currently on LLT and either an LDL-C level ≥55 mg/dL and a history of ASCVD or an LDL-C level of ≥70 mg/dL without a history of ASCVD	To receive enlicitide 20 mg qd or placebo	Mean percentage change in LDL-C level at week 24	The mean percentage change in LDL-C level at week 24 was −58.2% in the enlicitide group vs. 2.6% in the placebo group (between-group difference, −59.4% (95% CI: −65.6% to −53.2%; *p* < 0.001). Furthermore, significant reduction in non-HDL-C (between-group difference, −53.0%), apolipoprotein B (between-group difference, −49.1%), and lipoprotein(a) (between-group difference, −27.5%) levels were noted	Among adults with HeFH, treatment with enlicitide was well tolerated and significantly reduced levels of LDL-C, apolipoprotein B, non-HDL-C, and lipoprotein(a)
ORION 1 trial (2017) [125]	501	Patients with high CV risk disease and elevated LDL-C levels	To receive 200, 300, or 500 mg of inclisiran vs. placebo or two doses (at days 1 and 90) 100, 200, or 300 mg of inclisiran vs. placebo	The change in LDL-C level from baseline to 180 days	At day 180, the least-squares mean reductions in LDL-C levels were 27.9 to 41.9% after a single dose of inclisiran and 35.5 to 52.6% after two doses (*p* < 0.001 for all comparisons vs. placebo)	Inclisiran was found to lower PCSK9 and LDL-C levels among patients at high CV risk who had elevated LDL-C levels
ORION-10 trial (2020) [126]	1561	Patients with ASCVD who had elevated LDL-C levels despite receiving statin therapy at the maximum tolerated dose	To receive inclisiran 284 mg or placebo, administered on day 1, day 90, and every 6 months over a period of 540 days	The change in LDL-C level from baseline to day 510 and the time-adjusted percentage change in LDL-C from baseline after day 90 and up to day 540	The percentage change in LDL-C from baseline to day 510 was −51.3% with inclisiran and +1.0% with placebo, resulting in a −52.3% difference between groups (CI 95%, −55.7 to −48.8; *p* < 0.001). The time-adjusted change in LDL-C from baseline after day 90 and up to day 540 was −51.3% for inclisiran and +2.5% for placebo, reflecting a −53.8% difference between groups (CI 95%, −56.2 to −51.3; *p* < 0.001)	Reductions in LDL cholesterol levels of approximately 50% were obtained with inclisiran, administered subcutaneously every 6 months. More injection-site adverse events occurred with inclisiran than with placebo
ORION-11 trial (2020) [126]	1617	Patients with ASCVD or an ASCVD risk equivalent who had elevated LDL-C levels despite receiving statin therapy at the maximum tolerated dose	To receive inclisiran 284 mg or placebo, administered on day 1, day 90, and every 6 months over a period of 540 days	The change in LDL-C level from baseline to day 510 and the time-adjusted percentage change in LDL-C from baseline after day 90 and up to day 540	The percentage change in LDL-C from baseline to day 510 was −45.8% with inclisiran and +4.0% with placebo, resulting in a −49.9% difference between groups (CI 95%, −51.3 to −46.6; *p* < 0.001). The time-adjusted change in LDL-C from baseline after day 90 and up to day 540 was −45.8% for inclisiran and +3.4% for placebo, reflecting a −49.2% difference between groups (CI 95%, −51.6 to −46.8; *p* < 0.001)	Reductions in LDL cholesterol levels of approximately 50% were obtained with inclisiran, administered subcutaneously every 6 months. More injection-site adverse events occurred with inclisiran than with placebo
ORION 3 trial (2023) [127]	382	Patients with high CV risk disease and elevated LDL-C levels who had completed the ORION 1 trial	To receive twice-yearly 300 mg of inclisiran or 140 mg evolocumab every 2 weeks for one year then transitioned to 300 mg inclisiran	The percentage of LDL-C change with inclisiran from the start of ORION-1 up to day 210 of the ORION 3 in the inclisiran-only arm	In the inclisiran-only arm, LDL-C was reduced by 47·5% (95% CI 50·7–44·3) at day 210 and sustained over 1440 days	Twice-yearly inclisiran provided sustained reductions in LDL-C and PCSK9 concentrations and was well tolerated over 4 years in the extension study
ORION 8 trial (2024) [128]	3274	Patients ASCVD, ASCVD risk equivalent or HeFH	To receive twice-yearly 300 mg of inclisiran	The proportion of patients achieving pre-specified LDL-C goal	With inclisiran, 78.4% [CI 95%: 76.8 to 80.0] of patients achieved pre-specified LDL-C goals and mean percentage change in LDL-C was −49.4% (CI 95%: −50.4, −48.3)	Inclisiran demonstrated consistent and effective LDL-C lowering with a favorable long-term safety and tolerability profile
ORION 13 (2025) [129]	13	Patients ≥12 to <18 years with diagnosis of HoFH and LDL-C levels (>130 mg/dL) on statin treatment	To receive either 300 mg of inclisiran sodium or placebo	The mean percentage change in LDL-C from baseline to day 330	The mean percentage change in LDL-C from baseline at day 330 was −21.6% (13.4%) in the inclisiran group and +11.7% (30.5%) in the placebo group, with a mean between-group difference of −33.3% (CI 95%, −59.2% to −7.3%)	Inclisiran was effective in lowering LDL-C in adolescents with HoFH and was well tolerated

PCSK9-i: proprotein convertase subtilisin/kexin type 9 inhibitor; LDL-C: low-density lipoprotein cholesterol; CAD: coronary artery disease; CVD: cardiovascular disease; ACS: acute coronary syndrome; HR: hazard ratio; CI: confidence interval; IVUS: Intravascular ultrasonography; HI: high-intensity; ASCVD: atherosclerotic cardiovascular disease; MACE: major adverse cardiovascular event; MI: Myocardial Infarction; NSTEMI: non–ST-segment–elevation myocardial infarction; HeFH: heterozygous familial hypercholesterolemia; HoFH: homozygous familial hypercholesterolemia.

**Table 2:** Main randomized trials of treatment with bempedoic acid in secondary prevention.

Trial, Year	Patients (*n*)	Population Characteristics	Randomized Arms	Primary Endpoint	Main Results	Conclusions
CLEAR Harmony trial (2019) [143]	2230	Patients with ASCVD, HF hypercholesterolemia, or both and LDL-C ≥70 mg/dL in maximally tolerated statin therapy with or without additional LLT.	To receive bempedoic acid or placebo for 52 weeks.	Safety.	Adverse events, 78.5% in the bempedoic acid group and 78.7% in the placebo group and serious adverse events, 14.5% and 14.0%, respectively.	Bempedoic acid added to maximally tolerated statin therapy did not lead to a higher incidence of overall adverse events than placebo and led to significantly lower LDL-C levels.
CLEAR WISDOM trial (2019) [145]	779	Patients with ASCVD, HF hypercholesterolemia or both and LDL-C ≥70 mg/dL in maximally tolerated statin therapy with or without additional LLT.	To receive bempedoic acid or placebo for 52 weeks.	Percent change from baseline in LDL-C level at week 12.	Bempedoic acid lowered LDL-C levels significantly more than placebo at week 12, −15.1% vs. 2.4%; difference, −17.4% [95% CI, −21.0% to −13.9%]; *p* < 0.001.	Among patients at high risk for CVD receiving maximally tolerated statins; the addition of bempedoic acid compared with placebo resulted in a significant lowering of LDL-C level over 12 weeks.
Ballantyne et al. trial (2020) [142]	301	Patients at high risk of CV due to ASCVD, HF hypercholesterolemia, or multiple CVD risk factors.	To receive fixed-dose combination, bempedoic acid 180 mg, ezetimibe 10 mg or placebo added to stable background statin therapy for 12 weeks.	Percent change from baseline in LDL-C level at week 12.	Fixed-dose combination lowered LDL-C −36.2%; placebo 1.8% (placebo-corrected difference −38.0%, *p* < 0.001); ezetimibe alone (−23.2%; *p* < 0.001); bempedoic acid alone (−17.2%; *p* < 0.001).	The bempedoic acid and ezetimibe fixed-dose combination significantly lowered LDL-C versus placebo or other oral monotherapies and had a favorable safety profile when added to maximally tolerated statin therapy in patients with hypercholesterolemia and high CVD risk.
CLEAR Outcomes trial (2023) [144]	13,970	Statin-intolerant patients at high risk for CVD.	To receive bempedoic acid or placebo.	A composite of MACEs (death from CV causes, nonfatal MI, nonfatal stroke, or coronary revascularization).	Bempedoic acid 11.7% vs. placebo 13.3%, *p* = 0.004; a composite of death from CV causes, non- fatal stroke, or nonfatal MI 8.2% vs. 9.5%, *p* = 0.006; fatal or nonfatal MI 3.7% vs. 4.8%, *p* = 0.002; and coronary revascularization 6.2% vs. 7.6%, *p* = 0.001.	Treatment with bempedoic acid was associated with a lower risk of MACEs.

ASCVD: atherosclerotic cardiovascular disease; CV: cardiovascular; CVD: cardiovascular disease; HF: heterozygous familial; LDL-C: low-density lipoprotein; LLT: lipid-lowering therapy; MACEs: major adverse cardiovascular events; MI: myocardial infarction.

**Table 3:** Main randomized trials of treatment with EPA, PUFA, and IPE in patients with CVD.

Trial, Year	Patients (*n*)	Population Characteristics	Randomized Arms	Primary Endpoint	Main Results	Conclusions
JELIS trial (2007) [152]	18,645	Patients with a total cholesterol of 6·5 mmol/L or greater	To receive EPA 1800 mg daily plus statin or statin only	A composite of major coronary event	In patients with a history of CAD who were given EPA treatment, major coronary events were reduced by 19% (secondary prevention subgroup: 158 [8.7%] in the EPA group vs. 197 [10.7%] in the control group; *p* = 0.048). In patients with no history of CAD, EPA treatment reduced major coronary events by 18%, but this finding was not significant (104 [1.4%] in the EPA group vs. 127 [1.7%] in the control group; *p* = 0.132)	EPA is a promising treatment for prevention of major coronary events, and especially non-fatal coronary events, in Japanese hypercholesterolemic patients
Nosaka et al. (2017) [153]	241	Patients with ACS	To receive EPA 1800 mg daily plus pitavastatin or pitavastatin	CV events occurring within 1 year	A primary endpoint event occurred in 11 patients (9.2%) in the EPA group and 24 patients (20.2%) in the control group (absolute risk reduction, 11.0%; HR, 0.42; 95% CI, 0.21 to 0.87; *p* = 0.02)	Early initiation of treatment with EPA combined with statin after successful primary PCI reduced CV events after ACS
STRENGHT trial (2020) [154]	13,078	Patients with high CV risk, hypertriglyceridemia, and low levels of HDL-C	To receive omega-3 plus usual background therapy or corn oil plus usual background therapy	A composite of CV death, nonfatal myocardial infarction, nonfatal stroke, coronary revascularization, or unstable angina	The primary end point occurred in 785 patients (12.0%) treated with omega-3 CA vs. 795 (12.2%) treated with corn oil (HR, 0.99 [95% CI, 0.90–1.09]; *p* = 0.84). A greater rate of gastrointestinal adverse events was observed in the omega-3 CA group (24.7%) compared with corn oil–treated patients (14.7%)	Among statin-treated patients at high CV risk, the addition of omega-3 CA, compared with corn oil, to usual background therapies resulted in no significant difference in a composite outcome of MACEs
RESPECT-EPA trial (2022) [155]	3884	Patients with stable CAD and a low EPA/AA ratio (<0.4)	To receive EPA or placebo	A composite of CV death, nonfatal MI, non-fatal ischemic stroke, unstable angina pectoris, and coronary revascularization	The primary end point occurred in 112 of 1225 patients (9.1%) and 155 of 1235 patients (12.6%) in the EPA and control group, respectively (HR, 0.79 [CI 95%, 0.62–1.00]; *p* = 0.055)	IPE treatment resulted in a numerically lower risk of CV events that did not reach statistical significance in patients with chronic CAD, a low EPA/AA ratio, and statin treatment
Roncaglioni et al. (2013) [156]	6244	Patients with multiple CV risk factors or atherosclerotic vascular disease but not MI	To receive n-3 fatty acids 1 g daily or placebo	A composite of death from CV causes or admission to the hospital for CV causes	The primary end point occurred in 1478 of 12,505 patients included in the analysis (11.8%), of whom 733 of 6239 (11.7%) had received n-3 fatty acids and 745 of 6266 (11.9%) had received placebo (adjusted hazard ratio with n-3 fatty acids, 0.97; 95% confidence interval, 0.88 to 1.08; *p* = 0.58)	In a large general-practice cohort of patients with multiple CV risk factors, daily treatment with n-3 fatty acids did not reduce CV mortality and morbidity
OMEMI trial (2021) [157]	1027	Patients with a recent acute MI	To receive 1.8 g n-3 PUFA (930 mg eicosapentaenoic acid and 660 mg docosohexaenoic acid) versus placebo (corn oil)	A composite of nonfatal MI, unscheduled revascularization, stroke, death, HHF after 2 years	The primary endpoint occurred in 108 (21.4%) patients on n-3 PUFA versus 102 (20.0%) on placebo (HR, 1.08 [CI 95%, 0.82–1.41]; *p* = 0.60)	Not detect reduction in clinical events in elderly patients with recent AMI who were treated with 1.8 g n-3 PUFAs daily for 2 years
REDUCE-IT trial (2018) [158]	8179	Patients with CV disease or with diabetes and other risk factors, who had been receiving statin therapy and with TG level of 135 to 499 mg/dL and a LDL-C level of 41 to 100 mg/dL	To receive 2 g of EPA twice daily or placebo	A composite of CV death, nonfatal MI, nonfatal stroke, coronary revascularization, or unstable angina	A primary end-point event occurred in 17.2% of the patients in the IPE group, as compared with 22.0% of the patients in the placebo group (HR, 0.75; CI 95%, 0.68 to 0.83; *p* < 0.001)	Among patients with elevated TG levels despite the use of statins, the risk of ischemic events, including CV death, was significantly lower among those who received 2 g of IPE twice daily than among those who received placebo

AA: arachidonic acid; ACS: acute coronary syndrome; CI: confidence interval; CVD: cardiovascular disease; EPA: eicosapentaenoic acid; HDL-C: high-density lipoprotein cholesterol; HHF: heart failure hospitalization; HR: hazard ratio; IPE: Icosapent ethyl acid; MACEs: major adverse cardiovascular events; MI: myocardial infarction; PUFA: n-3 polyunsaturated fatty acids; TG: triglyceride.

**Table 4:** Main randomized trials of treatment with colchicine in ACS.

Trial, Year	Patients (*n*)	Population Characteristics	Randomized Arms	Primary Endpoint	Main Results	Conclusions
Deftereos T. et al. (2015) [184]	151	Patients with STEMI less than 12 h from pain onset	To receive colchicine or placebo for 5 days	Area under the curve of CPK-MB concentration	The area under the creatine kinase-myocardial brain fraction curve was 3.144 (IQR, 1.754–6.940) ng·h^−1^·mL^−1^ vs. 6.184 (IQR, 4.456–6.980) ng·h^−1^·mL^−1^ (*p* < 0.001)	Results suggest a potential benefit of colchicine in STEMI
COLIN Trial (2016) [185]	44	Patient admitted with STEMI referred for PCI	To receive 1 mg colchicine daily for 1 month plus OMT or OMT alone	CRP peak value during the index hospitalization	There was no significant difference in mean CRP peak value between the colchicine and control groups (29.03 mg/L vs. 21.86 mg/L, respetively; *p* = 0.36)	The effect of colchicine on inflammation in the context of STEMI could not be demonstrated
Vaidya K. et al. (2018) [190]	80	Patient with ACS within 1 month	To receive either 0.5 mg/day plus OMT or OMT alone	Change in low attenuation plaque volume	Colchicine therapy significantly reduced low attenuation plaque volume (mean 15.9 mm^3^ [40.9%] vs. 6.6 mm^3^ [17.0%]; *p* < 0.008)	Low-dose colchicine therapy favorably modifies coronary plaque, independent of high-dose statin intensification therapy and substantial low-density lipoprotein reduction
COLCOT Trial (2019) [189]	4745	Patients recruited within 30 days after MI	To receive colchicine (0.5 mg daily) vs. placebo	A composite of CV death, resuscitated cardiac arrest, MI, stroke and urgent hospitalization for angina leading to coronary revascularization	The primary end point occurred in 5.5% of the patients in the colchicine group, as compared with 7.1% of those in the placebo group (HR, 0.77; CI 95%, 0.61 to 0.96; *p* = 0.02)	Among patients with a recent MI, colchicine at a dose of 0.5 mg daily led to a significantly lower risk of ischemic CV events than placebo
LoDoCo-MI Trial (2019) [186]	237	Patients admitted with MI	To receive colchicine (0.5 mg daily) vs. placebo	The proportion of patients with Hs-CRP ≥ 2 mg/L after 30 days of treatment	At 30-day follow-up, 44% of patients treated with colchicine had a CRP level ≥2 mg/L compared to 50% of those randomized to placebo (*p* = 0.35)	Treatment with low-dose colchicine was safe and well tolerated, but was not associated with a significantly increased likelihood of achieving a CRP level 2 mg/L or lower absolute levels of CRP 30 days after an acute MI
COLCHICINE-PCI trial (2020) [191]	400	Patients referred for coronary angiography with possible PCI	To receive colchicine 1.8 mg/die or placebo pre-PCI	A composite of death, nonfatal MI and target vessel revascularization at 30 days	The primary outcome of PCI-related myocardial injury did not differ between colchicine (n = 206) and placebo (n = 194) groups (57.3% versus 64.2%, *p* = 0.19). The composite outcome of death, nonfatal MI and target vessel revascularization at 30 days (11.7% versus 12.9%, *p* = 0.82)	Acute preprocedural administration of colchicine attenuated the increase in interleukin-6 and Hs-CRP concentrations after PCI when compared with placebo but did not lower the risk of PCI-related myocardial injury
COPS Trial (2020) [187]	795	Patients presented with ACS and had evidence of CAD after coronary angiography	To receive colchicine, 0.5 mg twice daily for the first month, 0.5 mg daily for 11 months vs. placebo	A composite of death of any cause, ACS, ischemia-driven urgent revascularization, non-cardioembolic ischemic stroke	24 events in the colchicine group compared with 38 events in the placebo group (*p* = 0.09, log-rank). There was a higher rate of total death (8 versus 1; *p* = 0.017, log-rank) and, in particular, non CV death in the colchicine group (5 versus 0; *p* = 0.024, log-rank). The rates of reported adverse effects were not different (colchicine 23.0% versus placebo 24.3%), and they were predominantly gastrointestinal symptoms (colchicine, 23.0% versus placebo, 20.8%)	The addition of colchicine to standard medical therapy did not significantly affect CV outcomes at 12 months in patients with ACS and was associated with a higher rate of mortality
COVERT-MI (2021) [188]	192	Patients admitted for a first STEMI referred for primary PCI	To receive 2 mg loading dose of colchicine followed by 0.5 mg twice a day vs. placebo	Evaluation of IS by cardiac magnetic resonance imaging at 5 days	At 5 days, the gadolinium Enhancement-defined IS did not differ between the colchicine and placebo groups with a mean of 26 IQR [16,17,18,19,20,21,22,23,24,25,26,27,28,29,30,31,32,33,34,35,36,37,38,39,40,41,42,43,44] vs. 28.4 IQR [14,15,16,17,18,19,20,21,22,23,24,25,26,27,28,29,30,31,32,33,34,35,36,37,38,39,40] g of LV mass, respectively (*p* = 0.87)	Oral administration of high-dose colchicine at the time of reperfusion and for 5 days did not reduce IS assessed by cardiac magnetic resonance imaging
COLOCT Trial (2024) [192]	128	Patients with ACS with lipid-rich plaque	To receive colchicine (0.5 mg once daily) vs. placebo	The change in the minimal fibrous cap thickness at 12 months	Compared with placebo, colchicine therapy significantly increased the minimal fibrous cap thickness 51.9 μm (95% CI, 32.8–71) vs. 87.2 μm (CI 95%, 69.9–104.5); difference −34.2%, CI 95%, 9.7 to 58.6; *p* = 0.006	Colchicine resulted in favorable effects on coronary plaque stabilization at OCT in patients with ACS
CLEAR Trial (2025) [193]	7062	Patients that had STEMI or NSTEMI infarction and underwent PCI.	To receive either colchicine or placebo and either spironolactone or placebo	A composite of CV death, stroke or unplanned ischemia-driven coronary revascularization	A primary-outcome event occurred in 322 of 3528 patients (9.1%) in the colchicine group and 327 of 3534 patients (9.3%) in the placebo group over a median follow-up period of 3 years (HR, 0.99; CI 95%, 0.85 to 1.16; *p* = 0.93)	Treatment with colchicine, when started soon after MI and continued for a median of 3 years, did not reduce the incidence of the composite primary outcome

ACS: acute coronary syndrome; CAD: coronary acute disease; CV: cardiovascular; CI: confidence interval; CPK-MB: creatine kinase-myocardial brain fraction; CRP: C-reactive protein; Hs-CRP: high sensitive C-reactive protein; IQR: interquartile range; IS: infarct size; MI: myocardial infarction; NSTEMI: Non-ST-elevation myocardial infarction; OCT: optical coherence tomography; OMT: optimal medical therapy; PCI: percutaneous coronary intervention; STEMI: ST-elevation myocardial infarction.

**Table 5:** Main randomized trials of treatment with colchicine in CCS.

Trial, Year	Patients (n)	Population Characteristics	Randomized Arms	Primary Endpoint	Main Results	Conclusions
Nidorf M. S. et al. (2013) [194]	532	Patients with stable CAD	To receive OMT plus 0.5 mg colchicine or OMT alone for 3 years	A composite incidence of ACS, out-of-hospital cardiac arrest, or non-cardioembolic ischemic stroke	The primary outcome occurred in 15 of 282 patients (5.3%) who received colchicine and 40 of 250 patients (16.0%) assigned no colchicine (HR, 0.33; 95% CI 0.18 to 0.59; *p* < 0.001)	Colchicine 0.5 mg/day administered in addition to statins and other standard secondary prevention therapies appeared effective for the prevention of CV events in patients with stable CAD
LoDoCo2 Trial (2020) [195]	5522	Patients with chronic CAD	To receive 0.5 mg colchicine daily vs. placebo	A composite of CV death, spontaneous MI, ischemic stroke, or ischemia-driven coronary revascularization	A primary end-point event occurred in 187 patients (6.8%) in the colchicine group and in 264 patients (9.6%) in the placebo group (incidence, 2.5 vs. 3.6 events per 100 person-years; HR, 0.69; CI 95%, 0.57 to 0.83; *p* < 0.001)	The risk of CV events was significantly lower among those who received 0.5 mg of colchicine once daily than among those who received placebo

CAD: coronary artery disease; CCS: chronic coronary disease; CI: confidence interval; CV: cardiovascular; HR: hazard ratio; MI: myocardial infarction; OMT: optical medical therapy.

**Table 6:** Main randomized trials of treatment with non-colchicine anti-inflammatory drugs.

Trial, Year	Patients (*n*)	Population Characteristics	Randomized Arms	Primary Endpoint	Main Results	Conclusions
Ridker P. M. et al. (2012) [205]	556	Patients with well-controlled Diabetes Mellitus and high CV risk	To receive canakinumab at doses 5, 15, 50, or 150 mg monthly or placebo for 4 months	Effect on HbA1c, serum glucose, insulin, LDL-C, HDL-C, CRP, IL-6, and Fibrinogen	Compared with placebo, canakinumab had modest but nonsignificant effects on the change in HbA1c, glucose, and insulin levels. No effects were seen for LDL-C, HDL-C, or non HDL-C. The median reductions in CRP at 4 months were 36.4%, 53.0%, 64.6%, and 58.7% for the 5-, 15-, 50-, and 150 mg canakinumab doses, respectively, compared with 4.7% for placebo (all *p* values 0.02). The median reductions in IL-6 at 4 months across the canakinumab dose range tested were 23.9%, 32.5%, 47.9%, and 44.5%, respectively, compared with 2.9% for placebo (all *p* 0.008), and the median reductions in fibrinogen at 4 months were 4.9%, 11.7%, 18.5%, and 14.8%, respectively, compared with 0.4% for placebo (all *p* ≤ 0.0001)	Canakinumab, significantly reduces inflammation without major effect on LDL-C or HDL-C
TETHYS trial (2017) [203]	84	Patients with STEMI	To received MTX or placebo	Primary outcome was IS determined by calculating the AUC for CK release	Patients given MTX and placebo exhibited, respectively, median AUC for CK-MB of 9803.4 and 8037.0 (*p* = 0.42); median AUC for troponin of 3691.1 and 2132.6 (*p* = 0.09)	MTX did not improve long-term CV outcomes and was associated with more adverse events compared to placebo
CANTOS trial (2017) [206]	10,061	Patients with previous MI and CRP value higher than 2 mg/L	To receive placebo or Canakinumab at doses 50, 150, 300 mg every 3 months or placebo	A composite of nonfatal MI, nonfatal stroke or CV death	The incidence rate for the primary end point was 4.50 events per 100 person-years in the placebo group, 4.11 events per 100 person-years in the 50 mg group, 3.86 events per 100 person-years in the 150 mg group, and 3.90 events per 100 person-years in the 300 mg group. The HRs as compared with placebo were as follows: in the 50 mg group, 0.93 (95% confidence interval [CI], 0.80 to 1.07; *p* = 0.30); in the 150 mg group, 0.85 (95% CI, 0.74 to 0.98; *p* = 0.021); and in the 300 mg group, 0.86 (95% CI, 0.75 to 0.99; *p* = 0.031)	Anti-inflammatory therapy targeting the IL-1β innate immunity pathway with canakinumab at a dose of 150 mg every 3 months led to a significantly lower rate of recurrent CV events than placebo, independent of lipid-level lowering
CIRT trial (2018) [202]	4786	Patients with previous MI or multivessel coronary disease with additionally T2DM or metabolic syndrome	To receive a low dose of MTX or placebo	A composite of non-fatal MI, nonfatal stroke or CV death	The original primary end point occurred in 170 patients in the MTX group and in 167 in the placebo group (incidence rate, 3.46 vs. 3.43 per 100 person-years; HR, 1.01; 95% CI, 0.82 to 1.25, *p* = 0.91)	Among patients with stable atherosclerosis, low-dose MTX did not reduce levels of IL-1β, IL-6, or CRP and did not result in fewer CV events than placebo
RESCUE trial (2021) [207]	264	Patients with 18 years or older, moderate-to-severe chronic kidney disease and CRP value higher than 2 mg/L	To receive 7.5, 15, 30 mg of Ziltivekimab every 4 weeks up to 24 weeks or placebo	Percentage change from baseline in Hs-CRP after 12 weeks of treatment	At 12 weeks after randomization, median high-sensitivity CRP levels were reduced by 77% for the 7.5 mg group, 88% for the 15 mg group, and 92% for the 30 mg group compared with 4% for the placebo group. The median pairwise differences in percentage change in Hs-CRP between the ziltivekimab and placebo groups, after aligning for strata, were −66.2% for the 7.5 mg group, −77.7% for the 15 mg group, and −87.8% for the 30 mg group (all *p* < 0·0001)	Ziltivekimab markedly reduced biomarkers of inflammation relevant to atherosclerosis
Ferrari A. et al. (2025) [201]	32	Patients after first anterior STEMI	To received LDE-MTX or placebo the main secondary endpoints were changes in LVEDV, other LVR parameters and IS	LVEDV assessed by cardiac magnetic resonance at 90 ± 7 days	There was no difference in LVEDV between groups; 158.2 mL ± 40.5 vs. 163.9 mL ± 62.5 (*p* = 0.41). Among other secondary endpoints, there appeared to be a greater reduction in IS (%LV) in favor of the LDE-MTX group (−3.9 ± 6.9 vs. −9.4 ± 8.4, *p* = 0.030)	In patients with STEMI, LDE-MTX appears to be safe but did not influence LVEDV and other LVR parameters, although it possibly reduced IS at 90 days

AUC: area under the curve; CRP: C-reactive protein; CK: creatin kinase; CV: cardiovascular; HS-CRP: High-Sensitivity C Reactive Protein; HbA1c: glycated hemoglobin; HDL-C: high-density lipoprotein; IL:interleukin; IS: infarct size; LDE-MTX: lipid nanoemulsion-methotrexate; HR: hazard ratio; LDL-C: low-density lipoprotein cholesterol; LV: left ventricle; LVEDV: left ventricle end-diastolic volume; LVR: left ventricular remodeling; MTX: methotrexate; STEMI: ST-elevation myocardial infarction; T2DM: Type 2 Diabetes Mellitus.

## Data Availability

All data underlying this article will be shared upon reasonable request to the corresponding author.

## Abbreviations

The following abbreviations are used in this manuscript:

AA	Arachidonic Acid
ACC	American College of Cardiology
ACS	Acute Coronary Syndrome
AGE	Advanced Glycation End products
AHA	American Heart Association
ASCVD	Atherosclerotic Cardiovascular Disease
BMI	Body Mass Index
CAD	Coronary Artery Disease
CABG	Coronary Artery Bypass Graft
CCS	Chronic Coronary Syndrome
CETP	Cholesteryl Ester Transfer Protein
CI	Confidence Interval
CPK-MB	Creatine Kinase-Myocardial brain fraction
CV	Cardiovascular
CVD	Cardiovascular Disease
DES	Drug Eluting Stent
EAS	European Atherosclerosis Society
eLDL-TG	Estimating Low-Density Lipoprotein-Triglycerides
EPA	Eicosapentaenoic Acid
ESC	European Society of Cardiology
FFAs	Free Fatty Acids
FPG	Fasting Plasma Glucose
GDMT	Guideline Directed Medical Therapy
GISE	Italian Society of Interventional Cardiology
HbA1c	Glycated Hemoglobin
HDL	High-Density Lipoprotein
HeFH	Heterozygous familial hypercholesterolemia
HMG Co-A	Hydroxymethylglutaryl-CoA
HoFH	Homozygous Familial Hypercholesterolemia
HR	Hazard Ratio
Hs-CRP	High-sensitivity C-Reactive Protein
ICAM-1	Intercellular Adhesion Molecule-1
IDL	Intermediate-Density Lipoprotein
IL	Interleukin
IPE	Icosapent Ethyl
IQR	Interquartile range
IS	Infarct Size
IVUS	Intravascular Ultrasound
LDE-MTX	Lipid Nanoemulsion-Methotrexate
LDL-C	Low-Density Lipoprotein
LDL-R	Low-Density Lipoprotein Receptors
LLT	Lipid-Lowering Therapy
LPL	Lipoprotein Lipase
Lp(a)	Lipoprotein A
LVR	Left Ventricular Remodeling
MACE	Major Adverse Cardiovascular Event
MCP	Monocyte Chemotactic Protein
MI	Myocardial Infarction
MMP	Matrix Metalloproteinases
MTX	Methotrexate
NADH/NADPH	Nicotinamide adenine dinucleotide reduced form/Nicotinamide adenine dinucleotide phosphate reduced
NET	Neutrophil Extracellular Traps
NIRS	Near-Infrared Spectroscopy
NLRP3	NACHT, LRR and PYD domains-containing protein 3
NPC1L1	Niemann-Pick C1-like protein 1
NSTEMI	No-ST-up Elevation Myocardial Infarction
NYHA	New York Heart Association
OCT	Optical Coherence Tomography
OGTT	Oral Glucose Tolerance Test
OMT	Optimal Medical Therapy
OR	Odds Ratio
PAI	Plasma fibrinogen Activator Inhibitor
PCI	Percutaneous Coronary Intervention
PCSK9-i	Proprotein Convertase Subtilisin/Kexin type 9 Inhibitor
PUFA	Polyunsaturated Fatty Acids
ROS	Reactive Oxygen Species
RR	Relative Risk
SIL2r	Soluble IL-2 receptor
SMCs	Smooth Muscle Cells
STEMI	ST-up Elevation Myocardial Infarction
T2DM	Type 2 Diabetes Mellitus
TG	Triglycerides
TGRLs	Triglyceride-Rich Lipoproteins
TNF	Tumor Necrosis Factor
TyG	Triglyceride-Glucose
VCAM-1	Vascular Cell Adhesion Molecule-1
VLDL	Very-Low-Density Lipoprotein
